# Cannabinoid receptor type 2 (CB2)-selective *N*-aryl-oxadiazolyl-propionamides: synthesis, radiolabelling, molecular modelling and biological evaluation

**DOI:** 10.1186/2191-2858-2-32

**Published:** 2012-10-15

**Authors:** Thomas Rühl, Winnie Deuther-Conrad, Steffen Fischer, Robert Günther, Lothar Hennig, Harald Krautscheid, Peter Brust

**Affiliations:** 1 Department of Neuroradiopharmaceuticals, Institute of Radiopharmacy, Research Site Leipzig, Helmholtz-Zentrum Dresden-Rossendorf e.V., Permoserstr. 15, Leipzig, 04318, Germany; 2 Institute of Organic Chemistry, Faculty of Chemistry and Mineralogy, Universität Leipzig, Johannisallee 29, Leipzig, 04103, Germany; 3 Institute of Inorganic Chemistry, Faculty of Chemistry and Mineralogy, Universität Leipzig, Johannisallee 29, Leipzig, 04103, Germany

**Keywords:** Cannabinoid receptors, Molecular modelling, Autoradiography, ^18^F labelling, Molecular imaging, PET, Neuroimaging

## Abstract

**Background:**

The endocannabinoid system is involved in many physiological and pathological processes. Two receptors (cannabinoid receptor type 1 (CB1) and type 2 (CB2)) are known so far. Many unwanted psychotic side effects of inhibitors of this system can be addressed to the interaction with CB1. While CB1 is one of the most abundant neuroreceptors, CB2 is expressed in the brain only at very low levels. Thus, highly potent and selective compounds for CB2 are desired. *N*-aryl-((hetero)aromatic)-oxadiazolyl-propionamides represent a promising class of such selective ligands for the human CB2. Here, a library of various derivatives is studied for suitable routes for labelling with ^18^F. Such ^18^F-labelled compounds can then be employed as CB2-selective radiotracers for molecular imaging studies employing positron emission tomography (PET).

**Results:**

By varying the *N*-arylamide substructure, we explored the binding pocket of the human CB2 receptor and identified 9-ethyl-9*H*-carbazole amide as the group with optimal size. Radioligand replacement experiments revealed that the modification of the (hetero)aromatic moiety in 3-position of the 1,2,4-oxadiazoles shows only moderate impact on affinity to CB2 but high impact on selectivity towards CB2 with respect to CB1. Further, we could show by autoradiography studies that the most promising compounds bind selectively on CB2 receptors in mouse spleen tissue. Molecular docking studies based on a novel three-dimensional structural model of the human CB2 receptor in its activated form indicate that the compounds bind with the *N*-arylamide substructure in the binding pocket. ^18^F labelling at the (hetero)aromatic moiety at the opposite site of the compounds via radiochemistry was carried out.

**Conclusions:**

The synthesized CB2-selective compounds have high affinity towards CB2 and good selectivity against CB1. The introduction of labelling groups at the (hetero)aromatic moiety shows only moderate impact on CB2 affinity, indicating the introduction of potential labelling groups at this position as a promising approach to develop CB2-selective ligands suitable for molecular imaging with PET. The high affinity for human CB2 and selectivity against human CB1 of the herein presented compounds renders them as suitable candidates for molecular imaging studies.

## Background

The role of the endocannabinoid system in specific CNS disorders is related to the regulation of the temporal dynamics of neurotransmitter release by the retrograde cannabinoid signalling network [1]. Mediated by the G protein-coupled cannabinoid receptors [2], the release of endogenous or the administration of exogenous ligands affects both the long-term synaptic plasticity as well as the short-term regulation of synaptic transmission [1,3]. Two types of specific cannabinoid receptors have been cloned so far, termed cannabinoid receptor type 1 (CB1) and type 2 (CB2) [2,4]. The existence of additional cannabinoid-binding receptors has been suggested [5,6]. In contrast to classical neurotransmitters, endocannabinoids function as retrograde synaptic messengers which are released from postsynaptic neurons, diffuse across synapses, activate CB1 on presynaptic axons and eventually suppress neurotransmitter release [7]. In addition, endocannabinoids and their receptors control the decision about survival or death of neuronal cells [8], such that the pharmacological manipulation of this system might provide either neuroprotective or pro-apoptotic effects. A therapeutic role of cannabinoids has also been suggested for mood disorders [9], traumatic brain injury [10,11] and tumour treatment [12,13]. The development of CB2-selective anticancer agents is regarded to be advantageous in light of unwanted central effects exerted by binding of those agents to CB1 [14].

CB1 is abundantly expressed in the central nervous system and has been thoroughly investigated on cellular [15] and functional levels [16,17] in the brain. In contrast to this, only few and, to some extent, ambiguous data are available regarding CB2 expression and function in the brain. Initial reports stated that CB2 is mainly expressed peripherally in the cells and organs of the immune system such as the B lymphocyte-enriched marginal zone of the spleen or the cortex of lymph nodes [4,18]. However, more recent studies have proven structural and functional CB2 expression in primed glial (as reviewed in [5]) or neural progenitor cells [19]. Moreover, CB2 mRNA was detected in mouse [20] and rat cerebellum [21]. It could be shown in immunostaining studies that CB2 protein is abundant under basal conditions in neuronal and glial processes in the cerebellum and hippocampus of mouse and rat [22-24]. As reviewed recently by Atwood and Mackie [25], a growing number of reports substantiate a neuronal expression of CB2 through immunohistochemical data. Thus, it has been described that the activation of microglia is paralleled by an increase in CB2 immunoreactivity in the injured brain [26]. For instance, significantly increased CB2 expression levels were observed in severe Alzheimer's disease [27]. This indicates that CB2 expressed in the cerebellum might be involved in the pathogenesis of various neurodegenerative disorders and therefore might represent an interesting target for the diagnostics and therapy of such diseases [14,28]. Consequently, detailed investigation of the functional changes of both receptor subtypes CB1 and CB2 is needed to achieve a better understanding of the endocannabinoid system and the effects of potential cannabinoid therapeutics in the normal and diseased human brain [29].

Despite numerous *in vitro* approaches to directly identify CB2 in the brain, a non-invasive and quantitative analysis of these receptors *in vivo* has not been reported to date, probably due to the lack of ligands applicable for molecular imaging approaches such as positron emission tomography (PET). Recently, in a microPET study in a rat model, Evens et al. [30] observed specific binding of the type 2 cannabinoid receptor PET tracer ^11^C]NE40. In this model, hCB2 receptors were locally overexpressed in the brain after stereotactic injection of an adeno-associated viral vector (AAV2/7) encoding hCB2R with a point mutation (Asp80Asn) in the right striatum, yet the quantitative analysis of these receptors in native brain tissue remains a challenge.

Although CB2 has been already mentioned as a valuable biomarker of, e.g. neuroinflammation [30], the quantitative imaging of this target requires PET radioligands with superior affinity and specificity towards the low-abundance cerebral CB2 [31]. The ^11^C]-methoxy-labelled potent inverse CB2 agonist Sch225336 [32] showed only poor brain uptake in mice under baseline conditions. Various ^18^F-fluoroethoxy- [33] and ^11^C]-methoxy-labelled [34] 2-oxoquinoline derivatives were suggested as CB2 PET radioligands too, yet unfavourable brain-to-plasma ratios reflect the need for further improvement(s) regarding CB2 imaging in the healthy brain as recently reviewed [35].

## Methods

Here, we extend the synthesis of various CB2-binding [36]*N*-aryl-((hetero)aromatic-oxadiazolyl-propionamides as potential PET imaging compounds. Guided by molecular docking studies employing a novel comparative model of the human CB2 receptor, the promising site for ^18^F labelling was selected. *In vitro* binding experiments using human CB2-transfected Chinese hamster ovary (CHO) cells were performed. The obtained affinity data are in good agreement with predicted binding strength and could be verified by autoradiographic studies on mouse spleen slices.

## Results and discussion

### Synthesis of *N*-aryl-((hetero)aromatic)-oxadiazolyl-propionamides

**Scheme 1:**
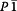
***N*-aryl-((hetero)aromatic)-oxadiazolyl-propionamides synthesized in this study.**

The substituted 1,2,4-oxadiazolyl-propionamides synthesized in this study are compiled in Scheme 1.

**Scheme 2:**
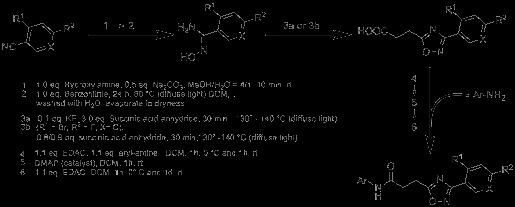
**Synthesis of *N*-aryl-((hetero)aromatic)-oxadiazolyl-propionamides.**

As depicted in Scheme 2, the two-step synthesis starts from a properly substituted nitrile and hydroxylamine yielding the hydroxy-amidin derivative. Following the route described by Cheng at al. [36], the products were dried after dilution with methylene chloride, and succinic acid anhydride was added to give the 3-phenyl-subsituted 1,2,4-oxadiazole acids. To support the formation of the oxadiazole acids, 0.1 eq. KF was added except for the compound with R^1^ = Br and R^2^ = F. In this case, the free 3-phenyl-substituted 1,2,4-oxadiazol-5-yl propanoic acid was protected, employing (diazomethyl)trimethylsilane. Subsequently, Br was substituted with CN by addition of 4.2 eq. KCN. Deprotection with LiOH yielded then the product. In a final step, the so obtained differently substituted 3-(hetero) aromatic-1,2,4-oxadiazol-5-yl-propanoic acids were coupled to various arylamides using standard (dimethylamino)-pyridine (DMAP)-catalysed carbodiimide chemistry. The obtained products were isolated either by semi-preparative high-performance liquid chromatography (HPLC) or crystallization (details can be found in the ‘Experimental’ section).

**Scheme 3:**
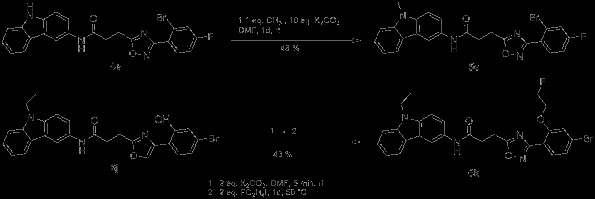
Two strategies to introduce fluorine into the molecule, allowing labelling with^18^F.

To suggest potential radiolabelling schemes of the compounds suitable for molecular imaging studies by PET, several strategies were evaluated, which are given in Scheme 3. For instance, **5e** was synthesized by methylation of **4e** with methyl iodide. This approach opens the way to introduce, for example, ^11^C or ^18^F by N-alkylation into the final compound. A further approach is the use of prosthetic groups, such as the introduction of fluorine-substituted alkyl chains via hydroxyl groups. In this study, **6k** was synthesized from **6j** by adding 1-fluoro-2-iodoethane. ^18^F can then be introduced into the molecule by using ^18^F-labelled alkylating agents. Another approach for the synthesis of ^18^F-labelled compounds is the replacement of fluorine with ^18^F via [2.2.2]cryptand chemistry, which is shown in this study as an example in the ‘Radiochemistry’ section.

**Figure 1:**
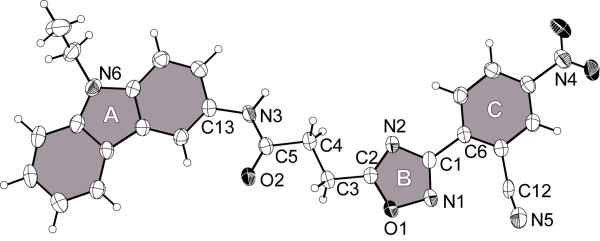
**X-ray crystal structure of 6f (50% thermal ellipsoids).** The planar ring systems have been noted by A, B and C and are highlighted in grey.

Figure 1 shows the single-crystal X-ray structure of compound **6f**. As expected, the amide bond of this molecule (C5-N3 in Figure 1) is in *trans* configuration. Though several bonds in **6f** are freely rotatable, the structure is planar. Thus, the dihedrals of the planes defined by the ring systems are 8.16(9)° between rings A and B and 11.2(1)° between rings B and C.

### *In vitro* binding studies

**Figure 2:**
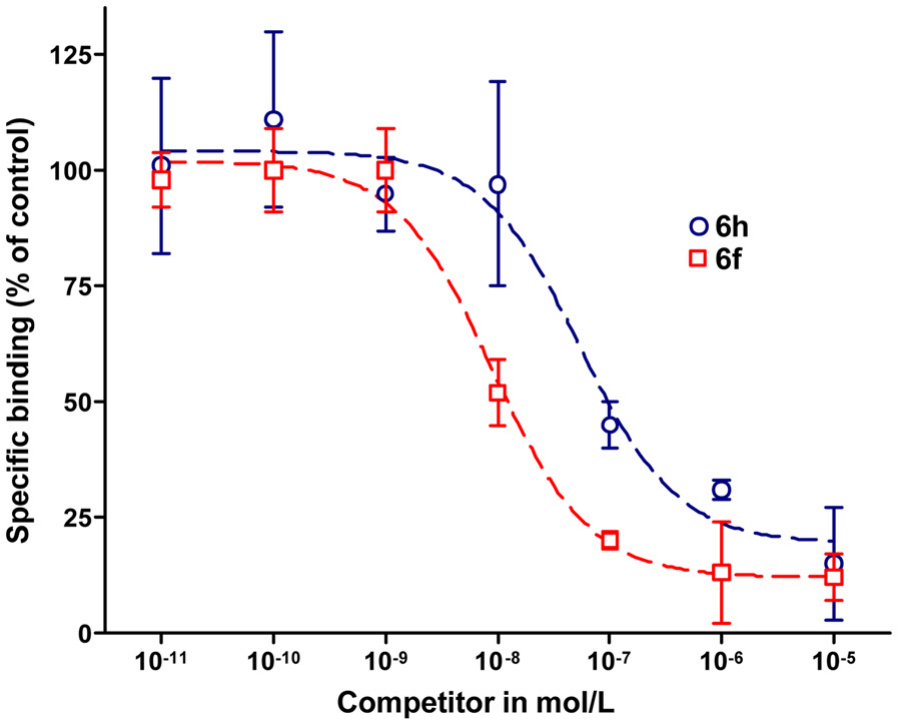
Radioligand displacement study of 6f and 6h against [^3^H]CP55,940 on membrane homogenates of hCB2-CHO cells.

The specific binding of ^3^H]CP55,940 towards cell membranes obtained from CHO cells stably transfected with human CB1 (hCB1-CHO) or CB2 (hCB2-CHO) hCB-CHO accounted for 40% to 50% of total binding. Non-specific binding was related mainly to binding to the glass fibre (data not shown). For the binding of ^3^H]CP55,940 towards hCB1-CHO and hCB2-CHO cell membranes, *K*_D_ values of 2.43 ± 1.89 nM (*n* = 3) and 1.48 ± 0.88 nM (*n* = 4) were determined in competitive binding experiments, which correspond with recently published data [37,38]. Kinetic analysis of the binding of ^3^H]CP55,940 towards hCB2-CHO cell membranes revealed the rate constants *k*_on_ = 0.266 × 10^9^ M^−1^ min^−1^ and *k*_off_ = 0.196 min^−1^. The accordingly estimated *K*_D_ value of 0.73 nM is consistent with the data obtained by saturation assay. The inhibition constants (*K*_i_) for each of the human CB receptors are compiled together with reference compounds CP55,940, SR144528, and SR141716A in Table 1. In Figure 2, the inhibition curves of **6f** and **6h** on the hCB2-CHO cell homogenates obtained in the radioligand displacement experiments with ^3^H]CP55,940 are given as examples.

**Table 1:** Affinities in nM and selectivity of the tested compounds towards hCB1 and hCB2 expressed in CHO cells

**Compound**	**hCB1**	**hCB2**	**Selectivity**^**a**^
**1a**	>10^5^	14.4	
**1b**	>10^5^	>10^5^	
**2a**	>10^5^	722	
**2b**	>10^5^	2,290	
**2c**	>10^5^	>10^5^	
**3b**	>10^5^	>10^5^	
**4e**	>10^5^	>10^5^	
**5e**	>10^5^	250	
**6a**	>10^5^	6.98	
**6b**	1,560	135	1.06
**6c**	>10^5^	1,920	
**6d**	7,470	12.5	2.78
**6e**	>10^5^	4.27	
**6f**	>10^5^	5.89	
**6g**	397	2.38	2.22
**6h**	>10^5^	39.4	
**6i**	>10^5^	>10^5^	
**6j**	>10^5^	5,710	
**6k**	>10^5^	3,190	
**6l**	>10^5^	223	
**6m**	>10^5^	>10^5^	
**7h**	6,010	>10^5^	
CP55,940	2.43	1.27	0.28
SR141716A	1.26	n.d.^b^	
SR144528	n.d.^b^	12.0	

^a^As calculated by p*K*_i_ (CB2) – p*K*_i_ (CB1) with pKi = -log (Ki); ^b^., not determined.

The data on the hCB2 affinity and selectivity of the synthesized compounds are in good agreement with the results published by Cheng et al. [36] and are consistent with the therein reported potencies of similar compounds. For compound **6a** (compound 44 in [36]), very high potency with an EC50 value of 0.85 nM was obtained by functional cAMP assays. Taken together with the high affinity measured for **6a** (*K*_i_ = 6.98 nM), it is obvious that the efficacy of this compound is mainly caused by the strength of the direct physical interaction with CB2. Thus, the *K*_D_ values can be seen as a direct measure of the activity of the compounds under investigation.

As the data in Table 1 show, the variation of the arylamide moiety has a significant impact on the affinity towards the cannabinoid receptors. The compounds with carbazolamides (Y^4^ in Scheme 1) show, on average, the highest affinity to hCB2. The 5-*tert*-butyl-isoxazol-3-yl derivative **1a** shows high affinity too, while a quinoline substitution (Y^2^ in Scheme 1) results in less favourable properties. The approximately 100-fold lower hCB2 affinity of the 3-substituted quinoline **2a** is in good agreement with published efficacy data, where a 6-substituted quinoline shows an approximately 50-fold lower [36] efficacy in comparison to **6a**. The situation is completely different for CB1. Here, replacement of the carbazole group with a smaller moiety results in a strong loss of affinity towards the hCB1 receptor. For compounds **2c**, **2b** and **2a**, binding on the hCB1 receptor could not be observed. Weak affinity towards hCB1 could be observed for **7h**, which bears a benzyl-substituted indole at the left-hand side. Remarkably, the affinity towards hCB2 is lost in comparison to **6h** despite the identical 2,4-diflourophenyl moieties at the 3-position of the oxadiazole ring. While the majority of the compounds bind to hCB2, those with an unsubstituted nitrogen in the ring system (aryles Y^1^, Y^2^, Y^4a^ in Scheme 1) show no binding to hCB1 at all. In general, the affinity of all compounds towards hCB1 is much lower than that towards hCB2. This might indicate a different binding mode of **6h** and **7h** at hCB2.

**Figure 3:**
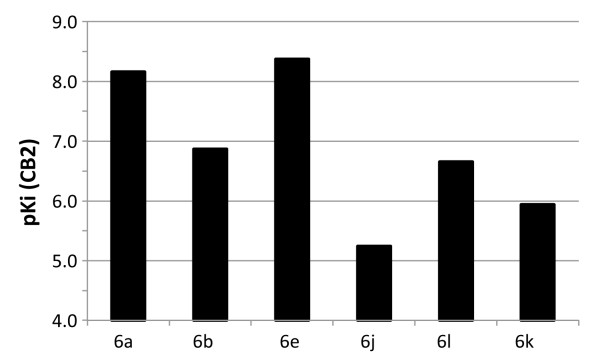
**Influence of substitution at ring C (*cf. *Figure**1**) on affinity towards hCB2.** The p*K*_i_ values were calculated according to p*K*_i_ = −log (*K*_i_). High p*K*_i_ values represent high affinity.

Comparing the compounds with the highest affinity (**6g**) and the best specificity for hCB2 (**6e**), the cause of this seems to be encoded in the functional group at *para*-position of the phenyl group. In concordance with published data [36], a fluorine atom at this position increases the affinity and efficacy towards hCB2. Depending on its electronegativity, a second substituent at *ortho*-position shows a significant impact on the affinity as shown in Figure 3. While the introduction of -Br (**6e**) or -CN (**6a**) leads to a higher affinity, the -OCH_3_ (**6l**) or -OCH_2_CH_2_F (**6k**) substituents decrease the affinity. Interestingly, replacement of the methoxy group with a hydroxyl moiety (**6j**) results in a further loss of affinity.

### Autoradiographic studies

**Figure 4:**
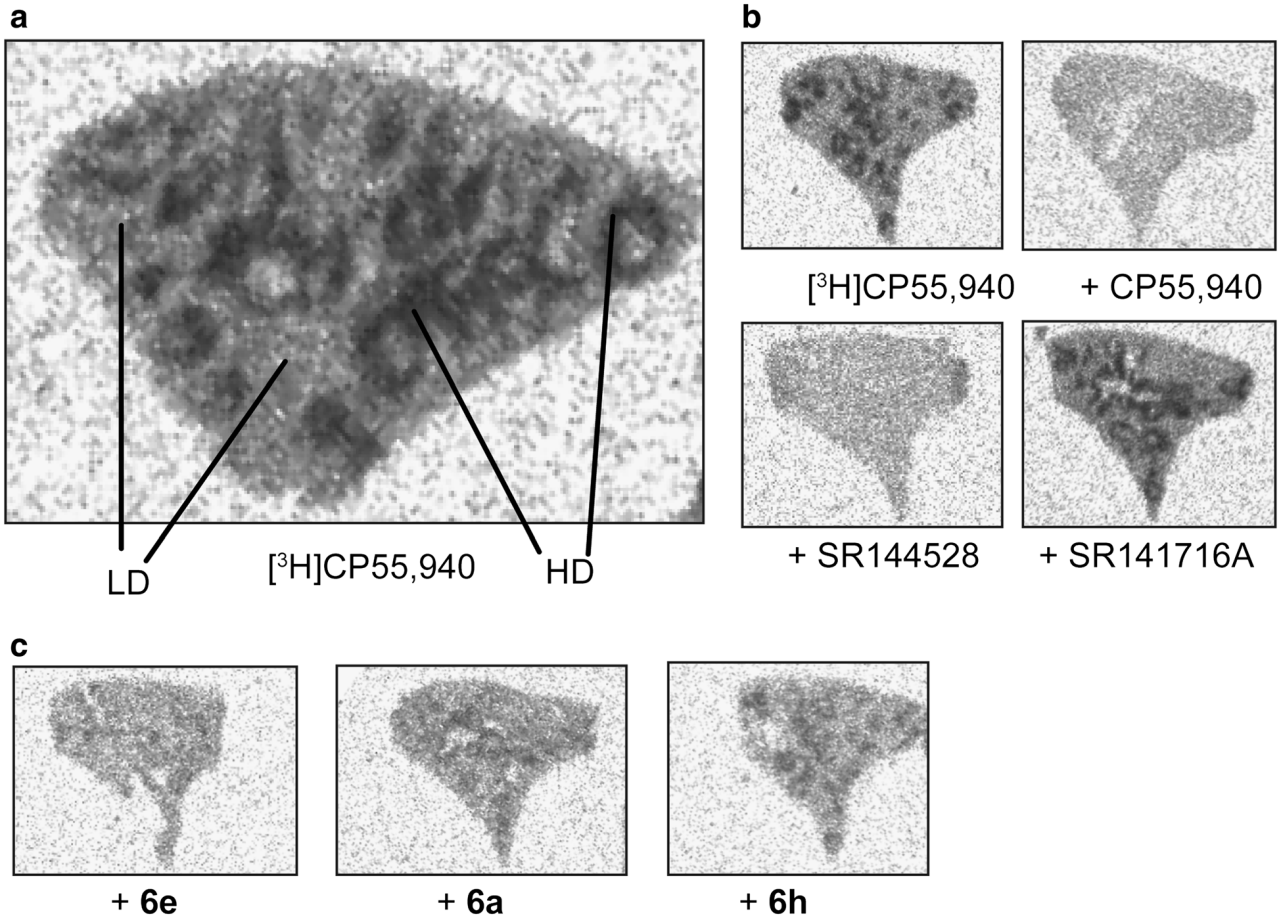
**Autoradiogram of representative coronal sections of CD-1 mouse spleen tissue. **(**a**) Incubated with 6 nM [^3^H]CP55,940. HD binding and LD binding represent the white and the red pulp, respectively (*cf.* text). (**b**) Incubation with 1 μM selective antagonists for CB2 (SR144528) and CB1 (SR141716A). (**c**) Compounds **6e**, **6a** and **6h** compete at 1 μM with the binding of [^3^H]CP55,940 on CB2 in good agreement with the determined binding data: **6e** >**6a** >**6h**.

For further investigation of the CB2 affinity of the test compounds, a preliminary autoradiographic investigation of the displacement potential of reference and test compounds on mouse spleen tissue co-incubated with ^3^H]CP55,940 has been performed. Figure 4 shows representative autoradiograms of coronal mouse spleen sections incubated with 6 nM ^3^H]CP55,940 in the presence of 1 μM of test compounds **6a**, **6e** and **6h**. For comparison, the autoradiograms for CP55,940, the CB1-selective antagonist SR141716A, and the CB2-selective antagonist SR144528 are shown in Figure 4b. As visible in Figure 4a, the distribution of ^3^H]CP55,940 was heterogeneous within the coronal spleen sections, showing a pattern similar to that reported previously [18,39]. Accordingly, high-density (HD) and low-density (LD) regions were assumed to reflect binding sites in the white or the red pulp, respectively.

To estimate the displacement efficacy of the reference and test compounds, the intensity of the radioligand binding in the HD regions was chosen to assess total binding of ^3^H]CP55,940. Specific binding of ^3^H]CPCP55,940 was calculated by subtracting the intensity of the homogenous binding of ^3^H]CP55,940 determined in the presence of 1 μM CP55,940 from the total binding values determined in the absence or presence of 1 μM compound. Accordingly, a high degree of specific binding of ^3^H]CP55,940 in mouse spleen has been identified, which accounts for approximately 90% of total ^3^H]CP55,940 binding. The density of specific binding sites of 6 nM ^3^H]CP55,940 in mouse spleen, estimated by converting the photostimulated luminescence per square millimetre values to femtomoles per milligram wet weight using ^3^H] standards, resembles, with 150 ± 25 fmol/mg wet weight, the values reported for the binding of 10 nM ^3^H]CP55,940 in rat spleen (59 to 108 fmol/mg wet weight) [18]. The assignment of the ^3^H]CP55,940 binding sites in mouse spleen to either CB1 or CB2 has been obtained by co-incubating the tissue with ^3^H]CP55,940 and either the CB1-selective SR141716A or the CB2-selective SR144,528 antagonist. As visible in the panels of Figure 4, co-incubation with 1 μM SR144528 displaced 78% of the specific binding of 6 nM ^3^H]CP55,940, while nearly no displacement has been detected in the presence of 1 μM SR141716A. In the latter, 94% of the specific CP55,940 binding remained. Thus, the basal binding of ^3^H]CP55,940 in mouse spleen was identified to reflect mainly CB2 binding, and the experimental conditions in this autoradiographic study are suitable to assess the CB2 binding potential of the test compounds.

At 1 μM, **6a**, **6e**, and **6h** displaced 83%, 71%, and 66% of the specific binding of 6 nM ^3^H]CP55,940 respectively, and these data correlate to the rank order of CB2 affinity obtained on hCB2-CHO cells (*K*_i_ = 4.27, 6.98 and 39.4 nM, respectively). However, the displacement potential of the three compounds in mouse spleen is slightly lower than that calculated according to the Gaddum equation, based on which a fractional occupancy of 98%, 97% and 83%, respectively, of ^3^H]CP55,940 binding sites has been estimated (*cf.* ‘Experimental’ section). This difference between experimental and calculated data might reflect the previously reported non-significant variances between affinity values obtained on native tissue by *in vitro* autoradiography and in radioligand displacement studies using transfected cells [40]. Alternatively, as shown for the binding of WIN55,212-2 towards ^3^H]CP55,940-labelled mouse or human CB2, species differences might also explain the slightly distinct affinity of the studied compounds [41].

### Molecular modelling

**Figure 5:**
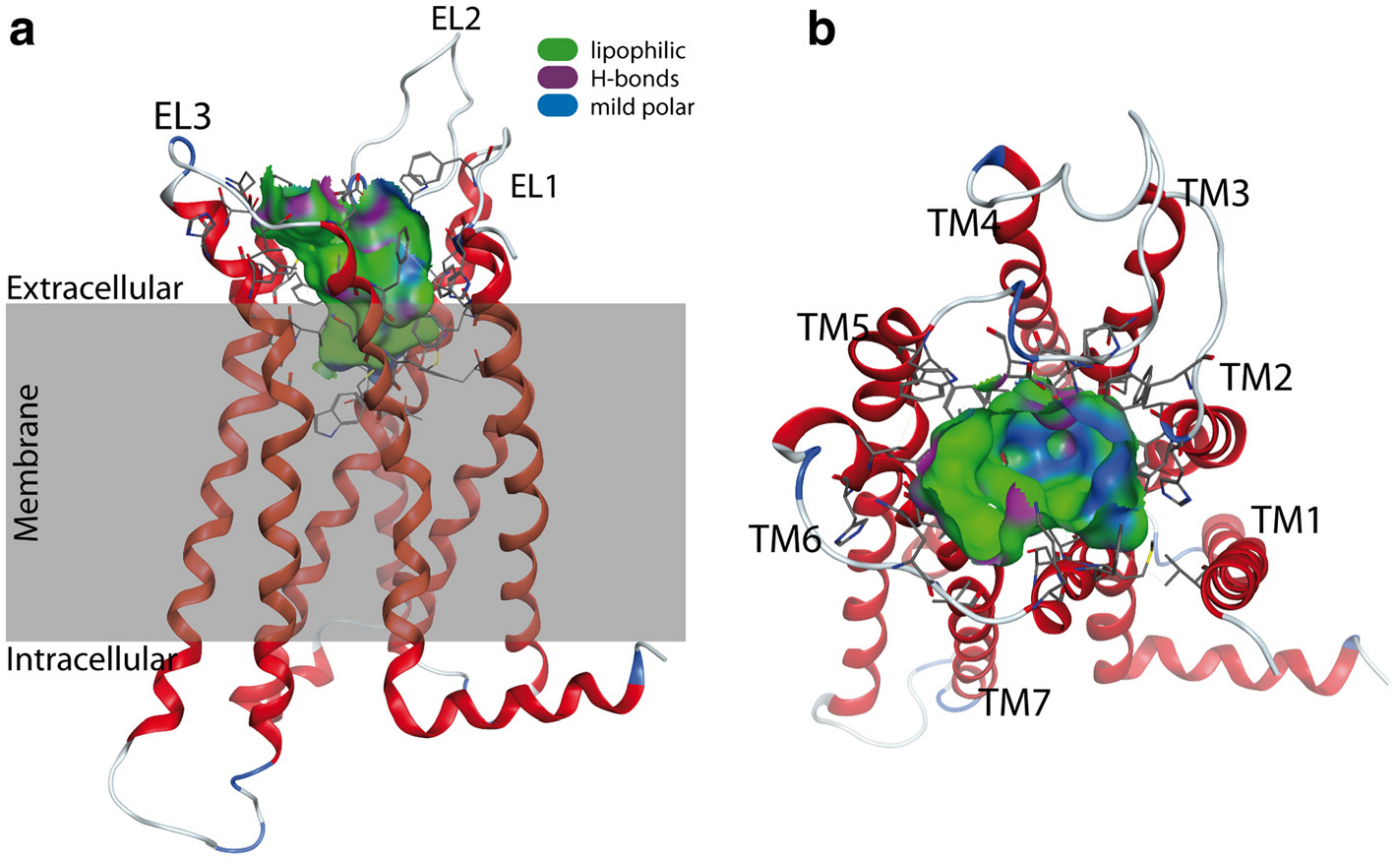
**Model of the human cannabinoid receptor type 2. **(**a**) Side view with the proposed transmembrane region shaded in grey. The protein is shown with the N-terminus at the top. (**b**) Top view from the extracellular space. The extracellular loops and the transmembrane helices are labelled to guide the reader. Only the residues forming the binding pocket are shown. The binding pocket identified by the Site Finder module of MOE (Chemical Computing Group Inc., Montreal, Canada) is shown as a surface with colour-coded features: H bonding (magenta), lipophilic (green), mild polar (blue).

In order to gain more insight into the binding of the herein synthesized compounds towards hCB2, we performed molecular modelling studies. As no experimentally determined structures of cannabinoid receptors are available yet, we created a three-dimensional (3D) structure model of this G protein-coupled receptor (GPCR) by comparative modelling. Based on the results obtained by a sequence search with the BLAST server, the 3D structure of the human beta1-adrenergic receptor (bAR1) [PDB:2Y00] [42] was identified as the structure with the highest sequence similarity to the hCB2 sequence. However, during the course of this study, a GPCR structure with a bound agonist was published [43]. In comparison to the bAR1 structure [PDB:2Y00], which is crystalized with a bound inverse agonist, this novel structure is more opened and thus represents a better description of a GPCR in an active state. Thus, the X-ray structure of the human A2A adenosine receptor with UK-432097 ([PDB:3QAK]) [43] was chosen as the template for comparative modelling. The co-crystalized ligand of this structure was kept in place during the building process of the receptor model to ensure a state ready to host an agonist. Figure 5 shows the constructed 3D model of the human CB2 with the proposed binding pocket. In agreement with mutagenesis data compiled by Poso and Huffman [44], residues known to be involved in the binding of CP55,940 are accessible in our model.

**Figure 6:**
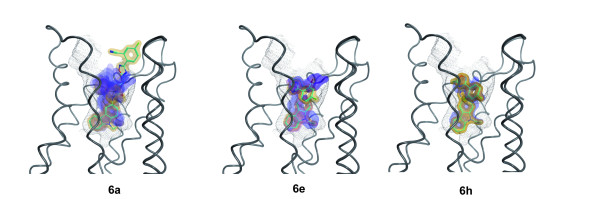
**Predicted binding modes of 6e, 6a, 6h to human CB2 model after docking with GOLD.** All compounds bind with their carbazole moiety (ring A, *cf.* Figure 1) in the binding pocket indicated by black dots. The figure was created with the software MOE (Chemical Computing Group Inc., Montreal, Canada) using the module PostDock [45]: The visibility of the surfaces is proportional to their binding energies based on Boltzman statistics. The surfaces are colour-coded with respect to the rmsd difference to the ligand with the lowest binding energy (from yellow to blue). As reference, the best-docked ligand is shown with cyan sticks.

The results of the docking of **6a**, **6e** and **6h** are shown in Figure 6. The pose with the lowest predicted binding energy is shown in green. In the majority of the predicted poses, the molecules are bent and deviated significantly from a planar structure, which was observed for **6f** in the single crystal. However, the bound conformation of a ligand is not necessarily identical to that of the free molecule in solution or in a packed crystal. Moreover, due to thermal effects, several differently populated conformations might exist. Typically, molecular docking procedures deliver multiple suggestions for the binding geometry of a ligand in a protein-ligand complex. Identifying the native pose out of these suggestions is often challenging, especially, if no experimentally determined reference structure is available. By taking into account the computed binding energy, it is possible to weight the suggested poses employing Boltzmann statistics.

**Figure 7:**
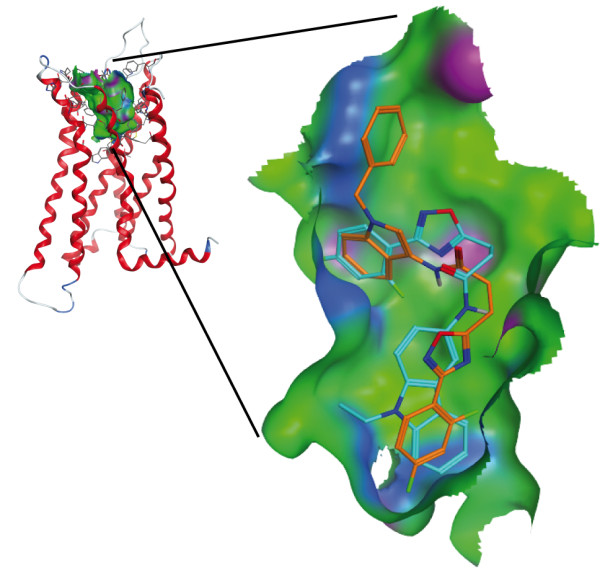
**Comparison of the predicted binding modes of 7h and 6h to the human CB2 model. 6h** (cyan) binds with the carbazole moiety in the binding pocket. For **7h** (orange), the preferred binding is with ring A pointing outside of the cavity. This figure was created using MOE (Chemical Computing Group Inc., Montreal, Canada).

In Figure 6 this is illustrated by encoding the energy in the visibility of the molecule according to the method implemented in the program PostDock [45]. While for **6h** only one representative pose was predicted, for **6a** and **6e**, two and three lower populated binding modes were found, respectively. This is illustrated by the blue shades in Figure 6, which represent the less favourable poses of these compounds. Nevertheless, in all cases, the three compounds bind primarily with their arylamide moiety inside the binding pocket. A detailed analysis of the binding site revealed a hydrophobic microdomain at the bottom of the pocket. The comparison of **6a** with **5e** indicates that a substituent at the N atom of the ring system increases the affinity towards CB2. If such a substituent is missing like in **4e**, no binding at hCB2 could be observed (data not shown). However, a replacement with a longer or bulkier moiety reduces the binding affinity too, as it is visible from the data obtained for **7h**. As illustrated in Figure 7, the preferred binding of **7h** is with the arylamide moiety outside the binding pocket, which, together with the determined binding data, substantiates a different binding mode of this compound. Thus, the 9-ethyl-9*H*-carbazole group seems to have an optimal size for fitting into the CB2 binding pocket.

The low nanomolar affinity of **6a** (*K*_i_ = 6.98 nM), **6e** (*K*_i_ = 4.27 nM) and **6h** (*K*_i_ = 39.4 nM) towards the human CB2 and the more than 1,000-fold selectivity over hCB1 (*K*_i_ > 1 μM) make these fluorine-substituted analogues the currently most promising candidates for further development of PET imaging agents within the series investigated. A proposed labelling at *p*-position of the phenyl group at the right-hand site seems to be favourable, e.g. the replacement of fluorine by ^18^F would allow applying these compounds for molecular imaging of CB2 receptors.

### Radiochemistry

**Scheme 4:**
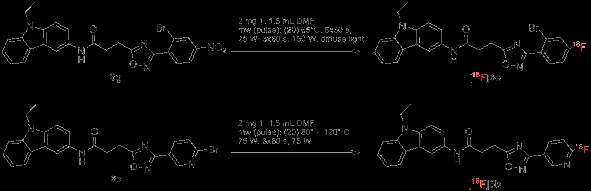
Radiochemistry.

The labelling with ^18^F was performed exemplarily on **6g**, yielding [^18^F]-**6e**, since **6e** has been identified as highly selective for hCB2 with a high affinity of *K*_i_ = 4.27 nM (Scheme 4). ^18^F was introduced by microwave-assisted nucleophilic substitution of the 4-NO_2_ moiety employing [2.2.2]cryptand (Kryptofix®, VWR International GmbH, Darmstadt, Germany). The electron-withdrawing effect (−I effect) of the Br substituent in *meta*-position to the leaving group -NO_2_ enables the nucleophilic attack.

However, this electron-withdrawing effect might not be strong enough as the reaction delivered only poor yields (3% radiochemical yield). An introduction of nitrogen into the aromatic ring should facilitate the nucleophilic substitution. Thus, the pyridine equivalent **6c** was labelled with ^18^F under comparable conditions (Scheme 4). Though not supported by a further electron-withdrawing substituent, the radiochemical yield is significantly higher with 28%. Unfortunately, compounds with a pyridine moiety as ring C show only low affinity towards hCB2, as visible in Table 1 (compounds **1b**, **2b, 2c, 3b, 6b, 6c**).

## Experimental

### Chemistry

All reagents were commercially obtained^a^ and used without further purification unless otherwise stated. Anhydrous solvents were transferred via an oven-dried syringe or cannula. Flasks were oven-dried under vacuum and cooled under a constant stream of nitrogen. ^1^H, ^13^C, and ^19^F nuclear magnetic resonance (NMR) spectra were recorded on VARIAN GEMINI 2000 (200 MHz for ^1^H NMR, 50 MHz for ^13^C NMR, 188 MHz for ^19^F NMR; Palo Alto, CA, USA), VARIAN ‘MERCURY plus’ (300 MHz for ^1^H NMR, 75 MHz for ^13^C NMR, 228 MHz for ^19^F NMR) and VARIAN ‘MERCURY plus’ and BRUKER DRX-400 (400 MHz for ^1^H NMR, 100 MHz for ^13^C NMR, 367 MHz for ^19^F NMR; Billerica, MA, USA). The chemical shifts are reported relative to the residual solvent peak, which was used as an internal reference (chemical shifts in *δ* values, *J* in Hz). The following abbreviations were used to describe peak splitting patterns when appropriate: br = broad, s = singlet, d = doublet, t = triplet, q = quartet, m = multiplet, dd = doublet of doublet, ddd = triplet of doublet, pt = pseudo-triplet. High-resolution mass spectrometry (MS) was performed on a Bruker Daltonics APEX II FT-ICR, and low-resolved mass spectra (using electrospray ionization (ESI)) were recorded on a Bruker ESQUIRE. Reactions involving moisture-sensitive reactants were performed in oven-dried glassware under an atmosphere of nitrogen, reactants being added via a syringe. Flash column chromatography was performed on silica gel (VWR, 60 Å, 40 to 63 μm, VWR GmbH, Darmstadt, Germany) and analytical thin-layer chromatography (TLC) on pre-coated silica gel plates (Roth, 60 Å, F254, 0.25 mm, Carl Roth GmbH & Co. KG, Karlsruhe, Germany). HPLC was performed on silica gel (ReproSil-Pur 120 ODS3, C18, end-capped (endc.)) from JASCO (Jasco Labor- & Datentechnik GmbH, Groß-Umstadt, Germany) with a HPLC from Nordantec GmbH (Bremerhaven, Germany). All compounds were obtained with 95% purity.

### 3-(3-(2-Bromo-4-fluorophenyl)-1,2,4-oxadiazol-5-yl)propanoic acid (8) and 2-bromo-4-fluorobenzamide (9)

To a mixture of deoxygenated hydroxylamine hydrochloride (517 mg, 7.4 mmol, 1.0 eq.) and sodium carbonate (394 mg, 3.7 mmol, 0.5 eq.), methanol/water (4:1, 35 mL) was added via a cannula, and the reaction mixture was stirred for 10 min. 2-Bromo-4-fluorobenzonitrile (1,487 mg, 7.4 mmol, 1.0 eq.) was added, and the reaction mixture was stirred under diffuse light for 18 h at 80°C. The reaction mixture was diluted with dichloromethane (DCM), washed with water and evaporated to dryness after concentration of the solvent *in vacuo*. To the deoxygenated crude intermediate succinic acid anhydride (638 mg, 6.4 mmol, 0.9 eq.) was added, and the reaction mixture was stirred under diffuse light for 30 min at 140°C. The crude material was purified by column chromatography on silica gel (eluting with petrol spirit or petrol ether/ethyl acetate/acetic acid 1:1:0.01) and by crystallization at room temperature (DCM/methanol/water) to give **8** (566 mg, 1.8 mmol, 24% yield) and **9** (559 mg, 2.6 mmol, 35% yield). **8**: ^1^H NMR (400 MHz, CDCl_3_): *δ* (ppm) = 7.84 (dd, *J* = 6 Hz, *J* = 9 Hz, 1H, CH), 7.47 (dd, *J* = 3 Hz, *J* = 8 Hz, 1H, CH), 7.14 (ddd, *J* = 3 Hz, *J* = 9 Hz, 1H, CH), 3.29 (t, *J* = 7 Hz, 2H, CH_2_), 3.01 (t, *J* = 7 Hz, 2H, CH_2_). ^19^F NMR (376 MHz, CDCl_3_): *δ* (ppm) = −107.89 (dd, *J* = 8 Hz, *J* = 14 Hz). ^13^C NMR (100 MHz, CDCl_3_): *δ* (ppm) = 177.7, 176.6, 167.2, 163.4 (d, *J* = 256 Hz), 133.3 (d, *J* = 9 Hz), 124.3 (d, *J* = 4 Hz), 122.8 (d, *J* = 10 Hz), 121.7 (d, *J* = 25 Hz), 114.9 (d, *J* = 22 Hz), 30.1, 21.7. High-resolution mass spectrometry (HRMS) (ESI, negative ion) *m*/*z*: 312.9, 314.9 [M-H]^−^. **9**: ^1^H NMR (400 MHz, CD_3_OD): *δ* (ppm) = 7.51 (dd, *J* = 6 Hz, *J* = 8 Hz, 1H, CH), 7.47 (dd, *J* = 3 Hz, *J* = 9 Hz, 1H, CH), 7.19 (pt, *J* = 2 Hz, *J* = 8 Hz, 1H, CH). ^19^F NMR (376 MHz, CD_3_OD): *δ* (ppm) = −111.64 (dd, *J* = 8 Hz, *J* = 15 Hz). ^13^C NMR (101 MHz, CD_3_OD): *δ* (ppm) = 172.4, 164.2 (d, *J* = 252 Hz), 136.2 (d, *J* = 4 Hz), 131.5 (d, *J* = 9 Hz), 121.5 (d, *J* = 25 Hz), 121.0 (d, *J* = 10 Hz), 115.7 (d, *J* = 22 Hz). HRMS (ESI, positive ion) *m*/*z*: 217.96086 [M+H]^+^.

### 3-(3-(2-Bromo-4-nitrophenyl)-1,2,4-oxadiazol-5-yl)propanoic acid (10) and 2-bromo-4-nitrobenzamide (11)

The propanoic acid **10** was synthesized similar to the synthesis of **8** but with 2-bromo-4-nitrobenzonitrile as the reacting agent. The mixture was diluted with ethyl acetate, and potassium fluoride (2 mg, 34 μmol, 0.1 eq.) was added during the reaction with succinic acid anhydride. The reaction mixture was stirred under diffuse light for 30 min at 130°C. For the purification, petrol spirit or petrol ether/ethyl acetate/acetic acid (1:1:0.005) was used, resulting in **10** (24 mg, 70 μmol, 16% yield) and **11** (22 mg, 90 μmol, 20% yield). **10**: ^1^H NMR (300 MHz, CDCl_3_/CD_3_OD 1:1): *δ* (ppm) = 8.56 (d, *J* = 2 Hz, 1H, C*H*), 8.29 (dd, *J* = 2 Hz, *J* = 9 Hz, 1H, C*H*), 7.89 (d, *J* = 9 Hz, 1H, C*H*). ^13^C NMR (75 MHz, CDCl_3_/CD_3_OD 1:1): *δ* (ppm) = 1,178.9, 173.0, 166.0, 148.5, 133.5, 132.2, 128.5, 122.0, 121.7, 29.4, 21.3. HRMS (ESI, negative ion) *m*/*z*: 339.95731 [M-H^+^]^−^, 680.92128 [2M-H^+^]^−^. **11**: ^1^H NMR (300 MHz, CDCl_3_/CD_3_OD): *δ* (ppm) = 8.50 (d, *J =* 2 Hz, 1H, C*H*), 8.28 (dd, *J =* 2 Hz, *J =* 8 Hz, 1H, C*H*), 7.68 (d, *J =* 8 Hz, 1H, C*H*). ^13^C NMR (100 MHz, CDCl_3_/CD_3_OD): *δ* (ppm) = 171.4, 150.0, 145.7, 130.6, 129.1, 123.7, 120.7. HRMS (ESI, positive ion) *m*/*z*: 244.95569 [M+H]^+^.

### 3-(3-(2-Fluoro-4-nitrophenyl)-1,2,4-oxadiazol-5-yl)propanoic acid (12) and 2-fluoro-4-nitrobenzamide (13)

Compounds **12** and **13** were prepared using 2-fluoro-4-nitrobenzonitrile (prepared as in the synthesis for **10**): **12**: 807 mg, 2.9 mmol, 10% yield; **13**: 93 mg, 0.5 mmol, 2% yield. **12**: ^1^H NMR (300 MHz, CDCl_3_): *δ* (ppm) = 8.26 (dd, *J =* 7 Hz, *J =* 8 Hz, 1H, C*H*), 8.06 to 8.18 (m, 2H, 2xC*H*), 3.32 (t, *J =* 7 Hz, 2H, C*H*_2_), 3.03 (t, *J =* 7 Hz, 2H, C*H*_2_). ^19^F NMR (282 MHz, CDCl_3_): *δ* (ppm) = −103.63 (dd, *J =* 7 Hz, *J =* 10 Hz). ^13^C NMR (75 MHz, CDCl_3_): *δ* (ppm) = 178.8, 176.0, 164.1 (d, *J =* 6 Hz), 160.5 (d, *J =* 263 Hz), 150.4 (d, *J =* 8 Hz), 131.8 (d, *J =* 3 Hz), 121.4 (d, *J =* 13 Hz), 119.5 (d, *J =* 4 Hz), 112.8 (d, *J =* 26 Hz), 30.7, 22.5. HRMS (ESI, negative ion) *m*/*z*: 280.03753 [M-H^+^]^−^, 316.01425 [M+Cl^−^]^−^, 561.08202 [2M-H^+^]^−^. **13**: ^1^H NMR (400 MHz, CD_3_OD): *δ* (ppm) = 8.18 to 8.07 (m, 2H, 2xC*H*), 7.97 (dd, *J =* 7 Hz, *J =* 8 Hz, 2H, C*H*_2_). ^19^F NMR (376 MHz, CD_3_OD): *δ* (ppm) = −112.07 (t, *J =* 8 Hz). ^13^C NMR (101 MHz, CD_3_OD): *δ* (ppm) = 166.9, 160.8 (d, *J =* 254 Hz), 151.5 (d, *J =* 9 Hz), 132.8 (d, *J =* 3 Hz), 130.1 (d, *J =* 15 Hz), 120.5 (d, *J =* 4 Hz), 113.1 (d, *J =* 29 Hz). HRMS (ESI, positive ion) *m*/*z*: 185.03583 [M+H]^+^, 207.01776 [M+Na]^+^, 391.04643 [2M+Na]^+^.

### 3-(3-(2-Bromo-4-methoxyphenyl)-1,2,4-oxadiazol-5-yl)propanoic acid (14) and 2-bromo-4-methoxybenzamide (15)

Compounds **14** and **15** were prepared using 2-bromo-4-methoxybenzonitrile (prepared as in the synthesis of **10**): **14**: 2539 mg, 7.8 mmol, 33% yield; **15**: impure. **14**: ^1^H NMR (300 MHz, CD_3_OD): *δ* (ppm) = 7.74 (d, *J =* 9 Hz, 1H, C*H*), 7.31 (d, *J =* 2 Hz, 1H, C*H*), 7.04 (dd, *J =* 3 Hz, *J =* 9 Hz, 1H, C*H*), 3.86 (s, 3H, C*H*_3_), 3.24 (t, *J =* 7 Hz, 2H, C*H*_2_), 2.92 (t, *J =* 7 Hz, 2H, C*H*_2_). Impurity: *δ* (ppm) = 2.56 (s, 1.0H). ^13^C NMR (76 MHz, CD_3_OD): *δ* (ppm) = 180.2, 175.1, 168.8, 163.2, 133.9, 123.6, 121.5, 120.6, 114.4, 56.3, 31.1, 22.9. Impurity: *δ* (ppm) = 176.2, 29.9. HRMS (ESI, negative ion) *m*/*z*: 324.98282 [M-H^+^]^−^, 650.97272 [2M-H^+^]^−^. **15**: ^1^H NMR (300 MHz, CD_3_OD): *δ* (ppm) = 7.44 (d, *J =* 9 Hz, 1H, C*H*), 7.19 (d, *J =* 3 Hz, 1H, C*H*), 7.00 (dd, *J =* 3 Hz, *J =* 9 Hz, 1H, C*H*), 3.83 (s, 3H, C*H*_3_). Impurity: *δ* (ppm) = 3.67 (s, 14.4H), 2.59 (s, 16H), 2.57 (s, 10H). HRMS (ESI, positive ion) *m*/*z*: 324.98282 [M-H^+^]^−^, 229.98094 [M+H]^+^.

### 3-(3-(2,4-Difluorophenyl)-1,2,4-oxadiazol-5-yl)propanoic acid (16)

Carboxylic acid **16** was prepared using 2,4-difluorobenzonitrile (prepared as in the synthesis for **10**): 5684 mg, 22.4 mmol, 64% yield. **16**: ^1^H NMR (300 MHz, CDCl_3_/CD_3_OD 1:1): *δ* (ppm) = 8.04 (dd, *J =* 8 Hz, *J =* 16 Hz, 1H, C*H*), 7.14 to 6.96 (m, 2H, 2xC*H*), 3.24 (t, *J =* 7 Hz, 2H, C*H*_2_), 2.92 (t, *J =* 7 Hz, 2H, C*H*_2_). ^19^F NMR (282 MHz, CDCl_3_/CD_3_OD 1:1): *δ* (ppm) = −104.91 to −105.25 (m, 1x*F*), −105.85 to −106.23 (m, 1x*F*). ^13^C NMR (75 MHz, CDCl_3_/CD_3_OD 1:1): *δ* (ppm) = 179.5, 174.2, 165.4 (d, *J =* 12 Hz, *J =* 254 Hz), 165.0 (d, *J =* 6 Hz), 161.9 (d, *J =* 12 Hz, *J =* 260 Hz), 132.8 (dd, *J =* 4 Hz, *J =* 10 Hz), 112.6 (dd, *J =* 4 Hz, *J =* 22 Hz), 112.1 (dd, *J =* 4 Hz, *J =* 13 Hz), 105.6 (pt, *J =* 26 Hz), 30.7, 22.5. HRMS (ESI, positive ion) *m*/*z*: 255.05735 [M+H]^+^, 277.03917 [M+Na]^+^, 531.09038 [2M+Na]^+^.

### 3-(3-(2-Cyano-4-fluorophenyl)-1,2,4-oxadiazol-5-yl)propanoic acid (17)

Lithium hydroxide monohydrate (50 mg, 1.200 mmol, 5.0 eq.) in water (1.2 mL) was added to **24** (66 mg, 241 μmol, 1.0 eq.) in methanol (9.5 mL) and stirred for 2.5 h at room temperature. Ethyl acetate (38 mL) and HCl solution (13 mL, 6 mol/L) were added, and the phases were mixed and separated. The aqueous HCl layer was washed with ethyl acetate, and the solvent was concentrated *in vacuo*. This material was used without further purification; **17**: 58 mg, 221 μmol, 92% yield. **17**: ^1^H NMR (300 MHz, CDCl_3_, 50°C): *δ* (ppm) = 8.51 (s broad, 1H, COO*H*), 8.15 (dd, *J =* 5 Hz, *J =* 9 Hz, 1H, C*H*), 7.53 (dd, *J =* 3 Hz, *J =* 8 Hz, 1H, C*H*), 7.42 (ddd, *J =* 3 Hz, *J =* 9 Hz, 1H, C*H*), 3.30 (t, *J =* 7 Hz, 2H, C*H*_2_), 3.01 (t, *J =* 7 Hz, 2H, C*H*_2_). ^19^F NMR (282 MHz, CDCl_3_, 50°C): *δ* (ppm) = −106.90 (dd, *J =* 8 Hz, *J =* 13 Hz). ^13^C NMR (75 MHz, CDCl_3_, 50°C): *δ* (ppm) = 178.9, 175.8, 165.6, 163.3 (d, *J =* 256 Hz), 132.3 (d, *J =* 9 Hz), 125.7 (d, *J =* 4 Hz), 121.8 (d, *J =* 25 Hz), 120.6 (d, *J =* 22 Hz), 116.0 (m), 113.3 (d, *J =* 9 Hz), 52.2, 30.0, 21.9. MS (ESI, positive ion) *m*/*z*: 284 [M+Na]^+^. MS (ESI, negative ion) *m*/*z*: 260 [M-H^+^]^−^.

### 3-(3-(2-Cyano-4-nitrophenyl)-1,2,4-oxadiazol-5-yl)propanoic acid (18)

The propanoic acid **18** was prepared using **26** (prepared as for the synthesis of **17**): 119 mg, 412 μmol, 99% yield. **18**: ^1^H NMR (300 MHz, CDCl_3_): *δ* (ppm) = 8.74 (d, *J =* 2 Hz, 1H, C*H*), 8.58 (dd, *J =* 2 Hz, *J =* 9 Hz, 1H, C*H*), 8.38 (d, *J =* 9 Hz, 1H, C*H*), 3.27 (t, *J =* 7 Hz, hidden by solvent, C*H*_2_), 2.93 (t, *J =* 7 Hz, 2H, C*H*_2_). ^13^C NMR (76 MHz, CDCl_3_): *δ* (ppm) = 182.1, 174.9, 166.5, 150.4, 135.4, 132.7, 131.1, 129.1, 116.8, 113.4, 30.9, 23.0. MS (ESI, negative ion) *m*/*z*: 287 [M-H^+^]^−^, 575[2M-H^+^]^−^.

### 3-(3-(4-Fluoro-2-hydroxyphenyl)-1,2,4-oxadiazol-5-yl)propanoic acid (19)

To a mixture of deoxygenated tribromoborane (7,528 mg, 30.1 mmol, 8.0 eq.) in dry DCM (130 mL), a mixture of **20** (1,000 mg, 3.8 mmol, 1.0 eq.) in dry DCM (60 mL) was added via a cannula at −70°C. The reaction mixture was stirred for 1 h at −70°C and overnight at room temperature. The reaction mixture was neutralized with saturated aqueous NaHCO_3_ solution at 1°C, washed with ethyl acetate, and evaporated to dryness after concentration of the solvent *in vacuo*. The crude material was purified by column chromatography on silica gel (eluting with DCM/MeOH = 9:1 and DCM/MeOH = 1:1). Water was added and the product was additionally purified by washing with DCM to give **19** (188 mg, 746 μmol, 20%). **19**: ^1^H NMR (400 MHz, CD_3_OD): *δ*_H_ (ppm) = 7.97 (pdd, *J* = 7 Hz, *J* = 9 Hz, 1H, C*H*), 6.79 to 6.71 (m, 2 H, 2xC*H*), 3.27 (t, *J* = 7 Hz, 2H, C*H*_2_), 2.94 (t, *J* = 7 Hz, 2H, C*H*_2_). ^19^F NMR (376 MHz, CD_3_OD): *δ*_F_ (ppm) = −109.06 to −109.20 (m). ^13^C NMR (101 MHz, CD_3_OD): *δ*_C_ (ppm) = 180.3, 175.1, 167.5, 166.9 (d, *J* = 250 Hz), 160.0 (d, *J* = 13 Hz), 131.6 (d, *J* = 11 Hz), 109.3 (d, *J* = 3 Hz), 108.5 (d, *J* = 23 Hz), 105.1 (d, *J* = 25 Hz), 31.0, 22.9. HR ESI -MS: C_11_H_9_FN_2_O_4_, *m*/*z* = 251.04731 [M-H^+^]^−^, 503.10122 [2M-H^+^]^−^.

### 3-(3-(4-Fluoro-2-methoxyphenyl)-1,2,4-oxadiazol-5-yl)propanoic acid (20)

Carboxylic acid **20** was prepared using 4-fluoro-2-methoxybenzonitrile (prepared as in the synthesis for **8**): 1,250 mg, 4.7 mmol, 15% yield. **20**: ^1^H NMR (400 MHz, CD_3_OD): *δ* (ppm) = 7.99 (dd, *J =* 7 Hz, *J =* 9 Hz, 1H, C*H*), 6.97 (dd, *J =* 2 Hz, *J =* 11 Hz, 1H, C*H*), 6.82 (ddd, *J =* 2 Hz, *J =* 8 Hz, *J =* 11 Hz, 1H, C*H*), 3.93 (s, 3H, C*H*_3_), 3.21 (t, *J =* 7 Hz, 2H, C*H*_2_), 2.82 (t, *J =* 7 Hz, 2H, C*H*_2_). Impurity: *δ* (ppm) = 3.16 (q, *J =* 7 Hz, 6.5H), 2.96 (s, 4.6H), 1.28 (t, *J =* 7 Hz, 10.2H). ^19^F NMR (376 MHz, CD_3_OD): *δ* (ppm) = −108.48 (td, *J =* 7 Hz, *J =* 11 Hz, *J =* 15 Hz). ^13^C NMR (101 MHz, CD_3_OD): *δ* (ppm) = 180.3, 177.0 (broad), 167.1, 166.9 (d, *J =* 250 Hz), 161.3 (d, *J =* 11 Hz), 133.8 (d, *J =* 11 Hz), 113.2 (d, *J =* 3 Hz), 108.3 (d, *J =* 22 Hz), 101.1 (d, *J =* 27 Hz), 56.7, 33.4 (broad), 22.1 (broad). Impurity: *δ* (ppm) = 177.6 (broad), 47.5, 23.8, 9.1. HRMS (ESI, negative ion) *m*/*z*: 265.06292 [M-H^+^]^−^, 531.13261 [2M-H^+^]^−^.

### 3-(3-(6-Bromopyridin-3-yl)-1,2,4-oxadiazol-5-yl)propanoic acid (21)

Compound **21** was prepared using 6-bromonicotinonitrile (prepared as in the synthesis for **8**): 164 mg, 550 μmol, 10% yield. **21**: ^1^H NMR (300 MHz, CDCl_3_/CD_3_OD 1:1, 30°C): *δ* (ppm) = 8.95 (dd, *J =* 1 Hz, *J =* 2 Hz, C*H*), 8.22 (dd, *J =* 2 Hz, *J =* 8 Hz, 1H, C*H*), 7.68 (dd, *J =* 1 Hz, *J =* 8 Hz, 1H, C*H*), 3.24 (t, *J =* 7 Hz, 2H, C*H*_2_), 2.92 (t, *J =* 7 Hz, 2H, C*H*_2_). ^13^C NMR (76 MHz, CDCl_3_/CD_3_OD 1:1, 30°C): *δ* (ppm) = 180.6, 174.1, 166.1, 149.2, 145.0, 137.9, 129.3, 123.3, 30.6, 22.5. HRMS (ESI, positive ion) *m*/*z*: 297.98160 [M+H]^+^, 319.96382 [M+Na]^+^, 616.93871 [2M+Na]^+^.

### 3-(3-(6-Fluoropyridin-3-yl)-1,2,4-oxadiazol-5-yl)propanoic acid (22)

Compound **22** was prepared using 6-fluoronicotinonitrile (prepared as in synthesis for **10**): 197 mg, 830 μmol, 5% yield. **22**: ^1^H NMR (400 MHz, CDCl_3_/CD_3_OD 1:1): *δ* (ppm) = 8.87 to 8.83 (m, 1 H, C*H*), 8.48 (ddd, *J =* 2 Hz, *J =* 7 Hz, *J =* 8 Hz, 1H, C*H*), 7.15 (ddd, *J =* 0.4 Hz, *J =* 3 Hz, *J =* 9 Hz, 1H, C*H*), 3.24 (t, *J =* 7 Hz, 2H, C*H*_2_), 2.93 (t, *J =* 7 Hz, 2H, C*H*_2_). ^19^F NMR (376 MHz, CDCl_3_/CD_3_OD 1:1): *δ* (ppm) = −65.88 (d, *J =* 6 Hz). ^13^C NMR (101 MHz, CDCl_3_/CD_3_OD 1:1): *δ* (ppm) = 180.5, 174.2, 166.0, 165.6 (d, *J =* 244 Hz), 147.4 (d, *J =* 15 Hz), 141.2 (d, *J =* 9 Hz), 122.2 (d, *J =* 5 Hz), 110.9 (d, *J =* 37 Hz), 30.6, 22.5. HRMS (ESI, negative ion) *m*/*z*: 236.04779 [M-H^+^]^−^, 272.02442 [M-Cl^−^]^−^, 473.10191 [2M-H^+^]^−^.

### Methyl 3-(3-(2-bromo-4-fluorophenyl)-1,2,4-oxadiazol-5-yl)propanoate (23)

(Diazomethyl)trimethylsilane (2 M in hexane, 1.3 mL, 2.6 mmol, 1.5 eq.) was added dropwise to deoxygenated **8** (547 mg, 1.7 mmol, 1.0 eq.) in dry toluene/methanol (9:1, 20 mL) at 0°C, and the reaction mixture was stirred for 30 min at room temperature. The crude product was purified by column chromatography on silica gel (eluting with petrol spirit or petrol ether/ethyl acetate 2:1) after concentration of the solvent *in vacuo* to give **23** (560 mg, 1.7 mmol, 98% yield). **23**: ^1^H NMR (400 MHz, CDCl_3_): *δ* (ppm) = 7.84 (dd, *J =* 6 Hz, *J =* 9 Hz, 1H, C*H*), 7.47 (dd, *J =* 3 Hz, *J =* 8 Hz, 1H, C*H*), 7.14 (ddd, *J =* 3 Hz, *J =* 9 Hz, 1H, C*H*), 3.73 (s, 3H, C*H*_3_), 3.29 (t, *J =* 7 Hz, 2H, C*H*_2_), 2.94 (t, *J =* 7 Hz, 2H, C*H*_2_). ^19^F NMR (376 MHz, CDCl_3_): *δ* (ppm) = −108.04 (dd, *J =* 8 Hz, *J =* 14 Hz). ^13^C NMR (101 MHz, CDCl_3_): *δ* (ppm) = 178.0, 171.6, 167.2, 163.3 (d, *J =* 256 Hz), 133.3 (d, *J =* 9 Hz), 124.5 (d, *J =* 4 Hz), 122.7 (d, *J =* 10 Hz), 121.6 (d, *J =* 25 Hz), 114.8 (d, *J =* 21 Hz), 52.1, 30.2, 22.0. MS (ESI, positive ion) *m*/*z*: 351 [M+Na]^+^.

### Methyl 3-(3-(2-cyano-4-fluorophenyl)-1,2,4-oxadiazol-5-yl)propanoate (24)

Copper(I) chloride (12 mg, 122 μmol, 0.4 eq.) was added to deoxygenated **10** (100 mg, 304 μmol, 1.0 eq.) in dry dimethylacetamide (2.2 mL). Potassium cyanide (79 mg, 1.215 mmol, 4.0 eq.) was added after 15 min, and the reaction mixture was stirred for 14 h at 130°C. Saturated ammonium chloride (2.2 mL), ethyl acetate (8.7 mL) and water (7.6 mL) were added, and the phases were mixed and separated. The aqueous layer was washed with ethyl acetate. The crude product was purified by column chromatography on silica gel (eluting with petrol spirit or petrol ether/ethyl acetate 2:1) after concentration of the solvent *in vacuo* to give **24** (23 mg, 83 μmol, 27% yield). **24**: ^1^H NMR (300 MHz, CDCl_3_): *δ* (ppm) = 8.15 (dd, *J =* 5 Hz, *J =* 9 Hz, 1H, C*H*), 7.53 (dd, *J =* 3 Hz, *J =* 8 Hz, 1H, C*H*), 7.42 (ddd, *J =* 3 Hz, *J =* 9 Hz, 1H, C*H*), 3.73 (s, 3H, C*H*_3_), 3.03 (t, *J =* 7 Hz, 2H, C*H*_2_), 2.96 (t, *J =* 7 Hz, 2H, C*H*_2_). ^19^F NMR (282 MHz, CDCl_3_): *δ* (ppm) = −106.85 (ddd, *J =* 5 Hz, *J =* 8 Hz). ^13^C NMR (100 MHz, CDCl_3_): *δ* (ppm) = 179.1, 171.6, 165.5, 163.2 (d, *J =* 256 Hz), 132.3 (d, *J =* 9 Hz), 125.6 (d, *J =* 4 Hz), 121.8 (d, *J =* 25 Hz), 120.6 (d, *J =* 21 Hz), 116.2 (d, *J =* 3 Hz), 113.0 (d, *J =* 10 Hz), 52.2, 30.1, 22.0. MS (ESI, positive ion) *m*/*z*: 276 [M+H]^+^, 298 [M+Na]^+^.

### Methyl 3-(3-(2-bromo-4-nitrophenyl)-1,2,4-oxadiazol-5-yl)propanoate (25)

Compound **25** was prepared using **10** (prepared as in the synthesis of **23**): 230 mg, 646 μmol, 98% yield. **25**: ^1^H NMR (400 MHz, CDCl_3_): *δ* (ppm) = 8.58 (d, *J =* 2 Hz, 1H, C*H*), 8.25 (dd, *J =* 2 Hz, *J =* 9 Hz, 1H, C*H*), 8.06 (d, *J =* 9 Hz, 1H, C*H*), 3.73 (s, 3H, C*H*_3_), 3.32 (t, *J =* 7 Hz, 2H, C*H*_2_), 2.96 (t, *J =* 7 Hz, 2H, C*H*_2_). ^13^C NMR (101 MHz, CDCl_3_): *δ* (ppm) = 178.7, 171.5, 166.5, 148.9, 133.9, 132.7, 129.2, 122.7, 122.1, 52.2, 30.1, 22.0. MS (ESI, positive ion) *m*/*z*: 378 [M+Na]^+^.

### Methyl 3-(3-(2-cyano-4-nitrophenyl)-1,2,4-oxadiazol-5-yl)propanoate (26)

The carboxylic acid **26** was prepared using **10** (prepared as for the synthesis of **24**): 83 mg, 275 μmol, 43% yield. **26**: ^1^H NMR (300 MHz, CDCl_3_): *δ* (ppm) = 8.68 (d, *J =* 2 Hz, 1H, C*H*), 8.54 (dd, *J =* 2 Hz, *J =* 9 Hz, 1H, C*H*), 8.39 (d, *J =* 9 Hz, 1H, C*H*), 3.73 (s, 3H, C*H*_3_), 3.34 (t, *J =* 7 Hz, 2H, C*H*_2_), 2.98 (t, *J =* 7 Hz, 2H, C*H*_2_). ^13^C NMR (75 MHz, CDCl_3_): *δ* (ppm) = 179.9, 171.5, 164.9, 148.7, 134.3, 131.3, 129.7, 127.4, 115.4, 112.7, 52.2, 30.0, 22.0. MS (ESI, positive ion) *m*/*z*: 303 [M+H]^+^, 325 [M+Na]^+^.

### General amide coupling method A

The coupling reaction was initiated by dropwise addition of *N*-ethyl-*N*′-(3-dimethylaminopropyl)carbodiimide (EDAC; 50 mg, 322 μmol, 1.1 eq.) in dry DCM (2.5 mL) to a mixture of 1.0 eq. of the deoxygenated propanoic acid derivate and 1.1 eq. amine in dry DCM (2.5 mL) at 0°C. The reaction mixture was stirred for 1 h at 0°C followed by stirring for 1 h at room temperature. A tip of spatula of 4-DMAP was added at 0°C, and the reaction mixture was stirred for 1 h at room temperature. EDAC (1.1 eq.) in dry DCM (2.5 mL) was added at 0°C, and the reaction mixture was again stirred for 14 h at room temperature followed by evaporation to dryness after concentration of the solvent *in vacuo*.

### 3-(3-(6-Bromopyridin-3-yl)-1,2,4-oxadiazol-5-yl)-*N*-(5-*tert*-butylisoxazol-3-yl)propanamide (1a)

Compound **1a** was prepared using **14** and 5-*tert*-butylisoxazol-3-amine according to method A. The crude material was purified by column chromatography on silica gel (elution with petrol spirit/ethyl acetate 2:1) and by HPLC (RP18, ReproSil-Pur 120 ODS3, endc., methanol/water = 85:15) and by crystallization at room temperature (methanol), giving **1a** (19 mg, 44 μmol, 27% yield). **1a**: ^1^H NMR (400 MHz, CDCl_3_): *δ* (ppm) = 10.20 (s broad, 1H, N*H*), 9.01 (d, *J =* 2 Hz, 1H, C*H*), 8.16 (dd, *J =* 2 Hz, *J =* 8 Hz, 1H, C*H*), 7.59 (d, *J =* 8 Hz, 1H, C*H*), 6.69 (s, 1H, C*H*), 3.39 (t, *J =* 7 Hz, 2H, C*H*_2_), 3.11 (t, *J =* 7 Hz, 2H, C*H*_2_), 1.31 (s, 9H, 3×C*H*_3_). ^13^C NMR (76 MHz, CDCl_3_): *δ* (ppm) = 182.2, 179.5, 168.9, 165.8, 158.0, 149.1, 145.0, 137.0, 128.5, 122.5, 93.6, 33.2, 32.5, 28.7, 22.1. HRMS (ESI, positive ion): C_17_H_18_BrN_5_O_3_, *m*/*z* = 420.06635 [M+H]^+^, 839.12559 [2M+H]^+^.

### 3-(3-(6-Fluoropyridin-3-yl)-1,2,4-oxadiazol-5-yl)-*N*-(5-*tert*-butylisoxazol-3-yl)propanamide (1b)

Compound **1b** was synthesized similar to **1a** with **22** (30 mg, 127 μmol, 1.0 eq.) as carboxylic acid component. Before evaporating, water (5.0 mL) was added, and the reaction mixture was washed with ethyl acetate. The crude material was purified as described for **1a**, giving **1b** (14 mg, 38 μmol, 30% yield). **1b**: ^1^H NMR (300 MHz, CD_3_OD): *δ*_H_ (ppm) = 8.85 (d, 1H, C*H*), 8.52 (ddd, *J* = 2 Hz, *J* = 8 Hz, *J* = 9 Hz, 1H, C*H*), 7.26 to 7.19 (m, 1H, C*H*), 6.54 (s, 1H, C*H*), 3.34 (t, *J* = 7 Hz, 2H, C*H*_2_), 3.06 (t, *J* = 7 Hz, 2H, C*H*_2_), 1.32 (s, 9H, 3×C*H*_3_). ^19^F NMR (282 MHz, CD_3_OD): *δ*_F_ (ppm) = −67.33 (d, *J* = 7 Hz). HR ESI +MS: C_17_H_18_FN_5_O_2_, *m*/*z* = 360.14680 [M+H]^+^, 382.12896 [M+Na]^+^, 719.28664 [2M+H]^+^, 741.26859 [2M+Na]^+^.

### 3-(3-(2-Cyano-4-fluorophenyl)-1,2,4-oxadiazol-5-yl)-*N*-(quinolin-3-yl)propanamide (2a)

Compound **2a** was prepared following general amide coupling method A with **17** and quinoline-3-amine. The crude material was purified by column chromatography on silica gel (elution with petrol spirit/ethyl acetate 1:1, ethyl acetate and DCM/methanol 1:1) and by crystallization at room temperature (acetonitrile), giving pure **2a** (25 mg, 64 μmol, 51% yield). **2a**: ^1^H NMR (300 MHz, CDCl_3_/CD_3_OD = 1:1, 50°C): *δ*_H_ (ppm) = 8.83 (d, *J* = 3 Hz, 1H, C*H*), 8.65 (d, *J* = 2 Hz, 1H, C*H*), 8.14 (dd, *J* = 5 Hz, *J* = 9 Hz, 1H, C*H*), 7.94 (pd, *J* = 8 Hz, 1H, C*H*), 7.77 (d, *J* = 1 Hz, *J* = 8 Hz, 1H, C*H*), 7.64 to 7.42 (m, 4H, C*H*), 3.41 (t, *J* = 7 Hz, 2H, C*H*_2_), 3.11 (t, *J* = 7 Hz, 2H, C*H*_2_). ^19^F NMR (282 MHz, CDCl_3_, 50°C): *δ*_F_ (ppm) = −107.89 (td, *J* = 5 Hz, *J* = 8 Hz, *J* = 16 Hz). APT NMR (75 MHz, CDCl_3_/CD_3_OD = 1:1, 50°C): *δ*_C_ (ppm) = 180.6, 171.2, 166.3, 164.1, (d, *J* = 255 Hz), 145.1, 144.9, 133.1, 133.0 (d, *J* = 9 Hz), 129.12, 129.08, 128.6, 128.3, 127.9, 127.6, 126.3 (*J* = 4 Hz), 125.2, 122.4 (d, *J* = 26 Hz), 121.5 (d, *J* = 22 Hz), 116.8 (d, *J* = 3 Hz), 113.4 (d, *J* = 9 Hz), 32.9, 22.5. HR ESI +MS: C_21_H_14_FN_5_O_2_, *m*/*z* = 388.12051 [M+H]^+^, 410.10243 [M+Na]^+^, 775.23385 [2M+H]^+^, 797.21557 [2M+Na]^+^.

### 3-(3-(6-Fluoropyridin-3-yl)-1,2,4-oxadiazol-5-yl)-*N*-(quinolin-3-yl)propanamide (2b)

The synthesis of **2b** followed the procedure described for **2a** with **21** as reactant. The crude material was purified under the same conditions as **2a**, resulting in 20 mg of **2b** (55 μmol, 44% yield). **2b**: ^1^ H NMR (400 MHz, CDCl_3_/CD_3_OD = 1:1): *δ*_H_ (ppm) = 8.86 to 8.79 (m, 2H, 2xC*H*), 8.68 (d, *J* = 2 Hz, 1H, C*H*), 8.46 (ddd, *J* = 2 Hz, *J* = 8 Hz, *J* = 8 Hz, 1H, C*H*), 7.93 (d, *J* = 8 Hz, 1H, C*H*), 7.77 (dd, *J* = 2 Hz, *J* = 8 Hz, 1H, C*H*), 7.65 to 7.58 (m, 1H, C*H*), 7.55 to 7.48 (m, 1H, C*H*), 7.12 (dd, *J* = 3 Hz, *J* = 9 Hz, 1H, C*H*), 3.34 (t, *J* = 7 Hz, 2H, C*H*_2_), 3.11 (t, *J* = 7 Hz, 2H, C*H*_2_). ^19^F NMR (376 MHz, CDCl_3_/CD_3_OD = 1:1): *δ*_F_ (ppm) = −66.00 (d, *J* = 7 Hz). ^13^C NMR (101 MHz, CDCl_3_/CD_3_OD = 1:1): *δ*_C_ (ppm) = 180.7, 171.3, 166.1, 165.6 (d, *J* = 244 Hz), 147.5 (d, *J* = 15 Hz), 145.0, 144.6, 141.2 (d, *J* = 9 Hz), 133.2, 129.19, 129.16, 128.44, 128.41, 128.1, 125.1, 122.3 (d, *J* = 5 Hz), 110.9 (d, *J* = 37 Hz), 32.9, 22.5. HR ESI +MS: C_19_H_14_FN_5_O_2_, *m*/*z* = 364.12066 [M+H]^+^, 386.10258 [M+Na]^+^, 727.23464 [2M+H]^+^, 749.21527 [2M+Na]^+^.

### 3-(3-(6-Bromopyridin-3-yl)-1,2,4-oxadiazol-5-yl)-*N*-(quinolin-3-yl)propanamide (2c)

Compound **2c** was synthesized by method A, by coupling **21** (50 mg, 168 μmol, 1.0 eq.) to quinolin-3-amine. Before evaporating to dryness, water (5.0 mL) was added to the reaction mixture and subsequently washed with ethyl acetate. The crude material was purified as described for **2a**, giving **2c** (33 mg, 77 μmol, 46% yield). **2c**: ^1^H NMR (400 MHz, CDCl_3_/CD_3_OD = 1:1): *δ*_H_ (ppm) = 8.94 (dd, *J* = 1 Hz, *J* = 2 Hz, 1H, C*H*), 8.78 (d, *J* = 2 Hz, 1H, C*H*), 8.70 (d, *J* = 2 Hz, 1H, C*H*), 8.18 (dd, *J* = 2 Hz, *J* = 8 Hz, 1H, C*H*), 7.93 (d, *J* = 8 Hz, 1H, C*H*), 7.76 (d, *J* = 1 Hz, *J* = 8 Hz, 1H, C*H*), 7.63 (dd, *J* = 1 Hz, *J* = 8 Hz, 1H, C*H*), 7.63 to 7.56 (m, 1H, C*H*), 7.53 to 7.47 (m, 1H, C*H*), 3.38 (t, *J* = 7 Hz, 2H, C*H*_2_), 3.10 (t, *J* = 7 Hz, 2H, C*H*_2_). ^13^C NMR (101 MHz, CDCl_3_/CD_3_OD = 1:1): *δ*_C_ (ppm) = 180.5, 170.8, 166.0, 149.1, 144.9, 144.7, 144.3, 137.7, 132.9, 129.1, 129.0, 128.9, 128.3, 128.2, 127.8, 124.8, 123.1, 32.6, 22.4. HR ESI +MS: C_19_H_14_BrN_5_O_2_, *m*/*z* = 424.04075 [M+H]^+^, 446.02267 [M+Na]^+^, 847.07404 [2M+H]^+^, 869.05547 [2M+Na]^+^.

### 3-(3-(6-Fluoropyridin-3-yl)-1,2,4-oxadiazol-5-yl)-*N*-(1-ethyl-1*H*-indol-5-yl)-propanamide (3b)

Compound **3b** was synthesized by coupling deoxygenated **21** (50 mg, 211 μmol, 1.0 eq.) to 1-ethyl-1*H*-indol-5-amine (37 mg, 232 μmol, 1.1 eq.) via method A. The crude material was purified by column chromatography on silica gel (elution with petrol spirit/ethyl acetate 1:1) and by crystallization at room temperature (DCM/petrol spirit), giving **3b** (61 mg, 162 μmol, 77% yield). **3b**: ^1^H NMR (300 MHz, CDCl_3_): *δ*_H_ (ppm) = 8.91 (d, *J* = 2 Hz, 1H, C*H*), 8.41 (dt, *J* = 2 Hz, *J* = 8 Hz, 1H, C*H*), 7.76 (s, 1H, C*H*), 7.61 (s broad, 1H, N*H*), 7.24 (d, *J* = 10 Hz, 1H, C*H*), 7.21 (d, *J* = 2 Hz, 1H, C*H*), 7.10 (d, *J* = 3 Hz, 1H, C*H*), 7.02 (dd, *J* = 3 Hz, *J* = 9 Hz, 1H, C*H*), 6.41 (d, *J* = 3 Hz, 1H, C*H*), 4.12 (q, *J* = 7 Hz, 2H, C*H*_2_), 3.38 (t, *J* = 7 Hz, 2H, C*H*_2_), 2.97 (t, *J* = 7 Hz, 2H, C*H*_2_), 1.43 (t, *J* = 7 Hz, 3H, C*H*_3_). ^19^F NMR (282 MHz, CDCl_3_): *δ*_F_ (ppm) = −64.21 (d, *J* = 5 Hz). ^13^C NMR (76 MHz, CDCl_3_): *δ*_C_ (ppm) = 179.8, 168.4, 165.6, 165.0 (d, *J* = 244 Hz), 147.4 (d, *J* = 16 Hz), 140.2 (d, *J* = 9 Hz), 133.5, 129.7, 128.8, 128.1, 121.5 (d, *J* = 5 Hz), 115.9, 113.2, 110.1 (d, *J* = 38 Hz), 109.5, 41.2, 33.0, 22.5, 15.6. HR ESI +MS: C_20_H_18_FN_5_O_2_, *m*/*z* = 380.15202 [M+H]^+^, 402.13403 [M+Na]^+^, 759.29638 [2M+H]^+^, 781.27720 [2M+Na]^+^.

### 3-(3-(2-Bromo-4-fluorophenyl)-1,2,4-oxadiazol-5-yl)-*N*-(9*H*-carbazol-3-yl)propanamide (4e)

Compound **4e** was prepared using **8** and 9*H*-carbazol-3-amine via coupling method A. The crude product was purified by column chromatography on silica gel (elution with petrol spirit/ethyl acetate 1:1, ethyl acetate and DCM/methanol 1:1) and by HPLC (RP18, ReproSil-Pur 120 ODS3, endc., methanol/water = 85:15) and by crystallization at room temperature (chloroform), giving 60 mg of **4e** (125 μmol, 64% yield). **4e**: ^1^H NMR (300 MHz, CD_3_OD): *δ* (ppm) = 8.26 (d, *J =* 2 Hz, 1H, C*H*), 7.96 (d, *J =* 8 Hz, 1H, C*H*), 7.82 (dd, *J =* 6 Hz, *J =* 9 Hz, 1H, C*H*), 7.55 (dd, *J =* 3 Hz, *J =* 9 Hz, 1H, C*H*), 7.49 to 7.29 (m, 4H, 4×C*H*), 7.21 (td, *J =* 2 Hz, *J =* 8 Hz, 1H, C*H*), 7.11 (t, *J =* 7 Hz, 1H, C*H*), 3.38 (t, *J =* 7 Hz, 2H, C*H*_2_), 3.04 (t, *J =* 7 Hz, 2H, C*H*_2_). ^19^F NMR (282 MHz, CD_3_OD): *δ* (ppm) = −110.02 (td, *J =* 6 Hz, *J =* 8 Hz). ^13^C NMR (76 MHz, CD_3_OD): *δ* (ppm) = 180.8, 171.4, 168.5, 164.8 (d, *J =* 254 Hz), 142.1, 138.7, 134.6 (d, *J =* 9 Hz), 131.1, 126.8, 126.1 (d, *J =* 4 Hz), 124.3, 124.2, 123.8 (d, *J =* 10 Hz), 122.4 (d, *J =* 25 Hz), 121.0, 120.7, 119.7, 116.0 (d, *J =* 22 Hz), 113.6, 111.8, 111.6, 33.4, 23.2. HR ESI +MS: C_23_H_16_BrFN_4_O_2_, *m*/*z* = 501.03343 [M+Na]^+^, 979.07777 [2M+Na]^+^.

### 3-(3-(2-Bromo-4-fluorophenyl)-1,2,4-oxadiazol-5-yl)-*N*-(9-methyl-9*H*-carbazol-3-yl)propanamide (5e)

Compound **5e** was synthesized by adding iodomethane (8 mg, 56 μmol, 1.3 eq.) in dry dimethylformamide (0.5 mL) dropwise to deoxygenated **4e** (21 mg, 43 μmol, 1.0 eq.) and potassium carbonate (59 mg, 430 μmol, 10.0 eq.). The reaction mixture was stirred for 20 h at room temperature followed by evaporation to dryness after concentration of the solvent *in vacuo*. The crude product was purified by hot filtration in methanol and by HPLC (RP18, ReproSil-Pur 120 ODS3, endc., acetonitrile/water = 85:15) with subsequent crystallization in the fridge (methanol) to give **5e** (8 mg, 17 μmol, 39% yield). **5e**: ^1^H NMR (300 MHz, CDCl_3_/CD_3_OD 1:1): *δ* (ppm) = 8.30 (d, *J =* 2 Hz, 1H, C*H*), 8.00 (d, *J =* 8 Hz, 1H, C*H*), 7.81 (d, *J =* 9 Hz, 1H, C*H*), 7.57 to 7.35 (m, hidden by solvent, 4×C*H*), 7.33 (d, *J =* 9 Hz, 1H, C*H*), 7.20 to 7.11 (m, 2 H, 2×C*H*), 3.81 (s, 3H, C*H*_3_), 3.40 (t, *J =* 7 Hz, 2H, C*H*_2_), 3.03 (t, *J =* 7 Hz, 2H, C*H*_2_). ^19^F NMR (282 MHz, CDCl_3_/CD_3_OD 1:1): *δ* (ppm) = −108.76 (td, *J =* 6 Hz, *J =* 8 Hz). HR ESI +MS: C_23_H_16_BrFN_4_O_2_, *m*/*z* = 515.04893 [M+Na]^+^, 1,007.10847 [2M+Na]^+^.

### 3-(3-(2-Cyano-4-nitrophenyl)-1,2,4-oxadiazol-5-yl)-*N*-(9-ethyl-9*H*-carbazol-3-yl)propanamide (6a)

For the synthesis of **6a**, **17** was coupled to 9-ethyl-9*H*-carbazol-3-amine as described in method A. After synthesis, water (5.0 mL) was added, and the reaction mixture was washed with ethyl acetate and evaporated to dryness after concentration of the solvent *in vacuo*. The crude product was purified by column chromatography on silica gel (elution with petrol spirit/ethyl acetate (2:1) + five drops of triethylamine and DCM/methanol (1:1) + five drops of triethylamine) and by crystallization at room temperature (DCM/methanol), giving pure **6a** (27 mg, 60 μmol, 50% yield). **6a**: ^1^H NMR (300 MHz, CDCl_3_/CD_3_OD = 1:1, 50°C): *δ*_H_ (ppm) = 8.28 (d, *J* = 2 Hz, 1H, C*H*), 8.13 (dd, *J* = 5 Hz, *J* = 9 Hz, 1H, C*H*), 7.99 (d, *J* = 8 Hz, 1H, C*H*), 7.54 (dd, *J* = 2 Hz, *J* = 8 Hz, 1H, C*H*), 7.52 to 7.37 (m, 3H, 3×C*H*), 7.35 (d, *J* = 8 Hz, 1H, C*H*), 7.31 (d, *J* = 9 Hz, 1H, C*H*), 7.13 (pt, *J* = 7 Hz, 1H, C*H*), 4.31 (q, *J* = 7 Hz, 2H, C*H*_2_), 3.40 (t, *J* = 7 Hz, 2H, C*H*_2_), 3.04 (t, *J* = 7 Hz, 2H, C*H*_2_), 1.36 (t, *J* = 7 Hz, 3H, C*H*_3_). ^19^F NMR (282 MHz, CDCl_3_/CD_3_OD = 1:1, 50°C): *δ*_F_ (ppm) = −107.74 (ps). ^13^C NMR (75 MHz, CDCl_3_/CD_3_OD = 1:1, 50°C): *δ*_C_ (ppm) = 179.6, 169.2, 165.1, 162.9 (d, *J* = 255 Hz), 140.1, 136.9, 131.9 (d, *J* = 9 Hz), 129.5, 125.32, 125.26 (d, *J* = 4 Hz), 122.5, 122.4, 121.3 (d, *J* = 26 Hz), 120.3 (d, *J* = 22 Hz), 119.9, 119.2, 118.2, 115.8, 112.4, 112.2, 108.1, 107.9, 37.0, 32.0, 21.9, 12.9. HR ESI +MS: C_26_H_20_FN_5_O_2_, *m*/*z* = 496.13901 [M+Na]^+^, 969.28932 [2M+Na]^+^.

### 3-(3-(6-Fluoropyridin-3-yl)-1,2,4-oxadiazol-5-yl)-*N*-(9-ethyl-9*H*-carbazol-3-yl)propanamide (6b)

Compound **6b** was synthesized as **6a** by coupling deoxygenated **22** with 9-ethyl-9*H*-carbazol-3-amine following method A. The crude material was purified by column chromatography on silica gel (elution with petrol spirit/ethyl acetate 1:1 and DCM/methanol 1:1) and by crystallization at room temperature (DCM/methanol), giving **6b** (42 mg, 98 μmol, 47% yield). **6b**: ^1^H NMR (400 MHz, CDCl_3_/CD_3_OD = 1:1): *δ*_H_ (ppm) = 8.83 (d, *J* = 2 Hz, 1H, C*H*), 8.47 to 8.38 (m, 1H, C*H*), 8.28 (d, *J* = 2 Hz, 1H, C*H*), 7.98 (d, *J* = 8 Hz, 1H, C*H*), 7.47 (dd, *J* = 2 Hz, *J* = 9 Hz, 1H, C*H*), 7.44 to 7.33 (m, 2H, 2×C*H*), 7.30 (d, *J* = 9 Hz, 1H, C*H*), 7.13 (t, *J* = 7 Hz, 1H, C*H*), 7.07 (dd, *J* = 2 Hz, *J* = 8 Hz, 1H, C*H*), 4.30 (q, *J* = 7 Hz, 2H, C*H*_2_), 3.36 (t, *J* = 7 Hz, 2 H, C*H*_2_), 3.03 (t, *J* = 7 Hz, 2H, C*H*_2_), 1.34 (t, *J* = 7 Hz, 3H, C*H*_3_). ^19^F NMR (282 MHz, CDCl_3_/CD_3_OD = 1:1): *δ*_F_ (ppm) = −65.79 (d, *J* = 7 Hz). ^13^C NMR (101 MHz, CDCl_3_/CD_3_OD = 1:1): *δ*_C_ (ppm) = 180.7, 170.3, 165.9, 165.5 (d, *J* = 244 Hz), 147.4 (d, *J* = 15 Hz), 141.1 (d, *J* = 9 Hz), 141.0, 137.8, 130.5, 126.4, 123.5, 123.3, 122.2 (d, *J* = 5 Hz), 120.9, 120.1, 119.2, 113.3, 110.8 (*J* = 37 Hz), 109.1, 109.0, 38.0, 32.8, 22.8, 14.0. HR ESI +MS: C_24_H_20_FN_5_O_2_, *m*/*z* = 452.14902 [M+Na]^+^, 859.32705 [2M+H]^+^, 881.30861 [2M+Na]^+^.

### 3-(3-(6-Bromopyridin-3-yl)-1,2,4-oxadiazol-5-yl)-*N*-(9-ethyl-9*H*-carbazol-3-yl)propanamide (6c)

Compound **6c** was prepared as described for **6b** with **21** as carboxylic acid reactant. The crude material was purified as described for **6b**, giving **6c** (42 mg, 86 μmol, 51% yield)*.***6c**: ^1^H NMR (300 MHz, CDCl_3_/CD_3_OD = 1:1): *δ*_H_ (ppm) = 8.93 (dd, *J* = 1 Hz, *J* = 2 Hz, 1H, C*H*), 8.28 (d, *J* = 2 Hz, 1H, C*H*), 8.16 (dd, *J* = 2 Hz, *J* = 8 Hz, 1H, C*H*), 7.97 (d, *J* = 8 Hz, 1H, C*H*), 7.60 (dd, *J* = 1 Hz, *J* = 8 Hz, 1H, C*H*), 7.47 (dd, *J* = 2 Hz, *J* = 9 Hz, 1H, C*H*), 7.44 to 7.33 (m, 2H, 2×C*H*), 7.30 (d, *J* = 9 Hz, 1H, C*H*), 7.17 to 7.09 (m, 1H, C*H*), 4.30 (q, *J* = 7 Hz, 2H, C*H*_2_), 3.36 (t, *J* = 7 Hz, 2H, C*H*_2_), 3.03 (t, *J* = 7 Hz, 2H, C*H*_2_), 1.35 (t, *J* = 7 Hz, 3H, C*H*_3_). ^13^C NMR (76 MHz, CDCl_3_/CD_3_OD = 1:1): *δ*_C_ (ppm) = 180.8, 170.2, 166.0, 149.1, 144.9, 141.0 137.77, 137.75, 130.5, 129.2, 126.4, 123.4, 123.3, 120.9, 120.0, 119.2, 113.3, 109.1, 109.0, 38.0, 32.8, 22.8, 14.0. HR ESI +MS: C_24_H_20_BrN_5_O_2_, *m*/*z* = 490.08770 [M+H]^+^, 512.06951 [M+Na]^+^, 979.16767 [2M+H]^+^, 1,001.14935 [2M+Na]^+^.

### 3-(3-(2-Fluoro-4-nitrophenyl)-1,2,4-oxadiazol-5-yl)-*N*-(9-ethyl-9*H*-carbazol-3-yl)propanamide (6d)

Compound **6d** was synthesized as **6a** using **12**. The crude product was purified by crystallization in the fridge (DCM), resulting in 69 mg of pure compound (145 μmol, 81% yield). **6d**: ^1^H NMR (400 MHz, CD_3_CN, 70°C): *δ* (ppm) = 8.39 (s broad, 1H, N*H*), 8.34 to 8.24 (m, 2H, 2×C*H*), 8.18 to 8.10 (m, 2H, 2xC*H*), 8.07 (d, *J =* 8 Hz, 1H, C*H*), 7.57 to 7.43 (m, 4H, 4xC*H*), 7.20 (t, *J =* 7 Hz, 1H, C*H*), 4.41 (q, *J =* 7 Hz, 2H, C*H*_2_), 3.40 (t, *J =* 7 Hz, 2H, C*H*_2_), 3.02 (t, *J =* 7 Hz, 2H, C*H*_2_), 1.39 (t, *J =* 7 Hz, 3H, C*H*_3_). ^19^F NMR (376 MHz, CD_3_CN, 90°C): *δ* (ppm) = −106.52 (s, broad). HR ESI +MS: C_25_H_20_FN_5_O_4_, *m*/*z* = 496.13901 [M+Na]^+^, 969.28932 [2M+Na]^+^.

### 3-(3-(2-Bromo-4-fluorophenyl)-1,2,4-oxadiazol-5-yl)-*N*-(9-ethyl-9*H*-carbazol-3-yl)propanamide (6e)

Compound **6e** was prepared as described for **6a** using **8** as carboxylic acid. The crude product was purified by column chromatography on silica gel (elution with DCM/methanol 100:1) and by crystallization at room temperature (DCM/methanol) to give **6e** (112 mg, 221 μmol, 76% yield) **6e**: ^1^H NMR (300 MHz, CDCl_3_): *δ* (ppm) = 8.26 (d, *J =* 2 Hz, 1H, C*H*), 8.02 (d, *J =* 8 Hz, 1H, C*H*), 7.83 (dd, *J =* 6 Hz, *J =* 9 Hz, 1H, C*H*), 7.69 (s broad, 1H, N*H*), 7.54 to 7.42 (m, 3H, 3xC*H*), 7.38 (d, *J =* 8 Hz, 1H, C*H*), 7.31 (d, *J =* 9 Hz, 1H, C*H*), 7.20 (pt, *J =* 8 Hz, 1H, C*H*), 7.12 (ddd, *J =* 2 Hz, *J =* 9 Hz, 1H, C*H*), 4.32 (q, *J =* 7 Hz, 2H, C*H*_2_), 3.45 (t, *J =* 7 Hz, 2H, C*H*_2_), 3.02 (t, *J =* 7 Hz, 2H, C*H*_2_), 1.40 (t, *J =* 7 Hz, 3H, C*H*_3_). ^19^F NMR (282 MHz, CDCl_3_): *δ* (ppm) = −107.95 (dd, *J =* 8 Hz, *J =* 14 Hz). ^13^C NMR (101 MHz, CDCl_3_): *δ* (ppm) = 178.6, 168.4, 167.2, 163.3 (d, *J =* 255 Hz), 140.4, 137.3, 133.3 (d, *J =* 9 Hz), 129.2, 125.9, 124.5 (d, *J =* 4 Hz), 123.0, 122.8 (d, *J =* 10 Hz), 122.7, 121.6 (d, *J =* 25 Hz), 120.6, 119.5, 118.8, 114.9 (d, *J =* 21 Hz), 113.0, 108.6, 108.5, 37.6, 33.1, 22.4, 13.78. HR ESI +MS: C_25_H_20_BrFN_4_O_2_, *m*/*z* = 507.08249 [M+H]^+^, 529.06444 [M+Na]^+^, 1013.15637 [2M+H]^+^, 1035.13966 [2M+Na]^+^.

### 3-(3-(2-Cyano-4-nitrophenyl)-1,2,4-oxadiazol-5-yl)-*N*-(9-ethyl-9*H*-carbazol-3-yl)propanamide (6f)

Compound **6f** was synthesized as **6a** using **18**. The crude product was purified by column chromatography on silica gel (elution with petrol spirit/ethyl acetate (2:1) + five drops of triethylamine and DCM/methanol (1:1) + five drops of triethylamine) and by crystallization at room temperature (DCM/methanol and acetonitrile), resulting in **6f** (25 mg, 51 μmol, 25%). **6f**: ^1^H NMR (300 MHz, CD_3_CN, 70°C): *δ*H (ppm) = 8.71 (d, *J* = 2 Hz, 1H, C*H*), 8.54 (dd, *J* = 2 Hz, J = 9 Hz, 1H, C*H*), 8.38 (d, *J* = 9 Hz, 1H, C*H*), 8.32 (ps, 1H, C*H*), 8.07 (pd, *J* = 8 Hz, 1H, C*H*), 7.54 (dd, *J* = 2 Hz, *J* = 9 Hz, 1H), 7.51 to 7.43 (m, 3H, 3×C*H*), 7.20 (pt, *J* = 7 Hz, 1H, C*H*), 4.41 (q, *J* = 7 Hz, 2H, C*H*_2_), 3.43 (t, *J* = 7 Hz, 2H, C*H*_2_), 3.04 (t, *J* = 7 Hz, 2H, C*H*_2_), 1.39 (t, *J* = 7 Hz, 3H C*H*_3_). HR ESI +MS: C_26_H_20_N_6_O_4_, *m*/*z* = 503.14351 [M+Na]^+^, 983.29765 [2M+Na]^+^.

### 3-(3-(2-Bromo-4-nitrophenyl)-1,2,4-oxadiazol-5-yl)-*N*-(9-ethyl-9*H*-carbazol-3-yl)propanamide (6g)

Compound **6g** was prepared as **6a** but with **10** as the reactant. The crude product was purified as described for **6e**, yielding **6g** (106 mg, 198 μmol, 68%). **6g**: ^1^H NMR (300 MHz, CDCl_3_, 50°C): *δ*_H_ (ppm) = 8.57 (d, *J* = 2 Hz, 1H, C*H*), 8.36 to 8.14 (m, 2H, 2×C*H*), 8.03 (pd, *J* = 8 Hz, 2H, 2×C*H*), 7.62 to 7.13 (m, hidden by solvent), 4.35 (q, *J* = 7 Hz, 2H, C*H*_2_), 3.49 (ps broad, 2H, C*H*_2_), 3.04 (ps broad, 2H, C*H*_2_), 1.43 (t, *J* = 7 Hz, 3H, C*H*_3_). HR ESI +MS: C_25_H_20_BrN_5_O_4_, *m*/*z* = 556.05940 [M+Na]^+^, 1,067.14721 [2M+H]^+^, 1,089.12816 [2M+Na]^+^.

### 3-(3-(2,4-Difluorophenyl)-1,2,4-oxadiazol-5-yl)-*N*-(9-ethyl-9*H*-carbazol-3-yl)propanamide (6h)

Compound **6h** was synthesized similar to the procedure described for **6a** using **16** as carboxylic acid. The crude product was purified by column chromatography on silica gel (elution with petrol spirit/ethyl acetate 2:1, petrol spirit/ethyl acetate 2:1 and DCM/methanol 1:1) and by crystallization at room temperature (DCM/methanol 1:1), yielding **6h** (39 mg, 88 μmol, 49%). **6h**: ^1^H NMR (400 MHz, CDCl_3_/CD_3_OD = 1:1): *δ*_H_ (ppm) = 8.29 (d, *J* = 2 Hz, 1H, C*H*), 8.04 (pdd, *J* = 8 Hz, *J* = 16 Hz, 1H, C*H*), 8.00 (d, *J* = 8 Hz, 1H, C*H*), 7.49 (dd, *J* = 2 Hz, *J* = 9 Hz, 1H, C*H*), 7.46 to 7.36 (m, 2H, 2xC*H*), 7.33 (d, *J* = 8 Hz, 1H, C*H*), 7.14 (pt, *J* = 6 Hz, 1H, C*H*), 7.06 to 6.97 (m, 2H, 2xC*H*), 4.33 (q, *J* = 7 Hz, 2H, C*H*_2_), 3.38 (t, *J* = 7 Hz, 2H, C*H*_2_), 3.03 (t, *J* = 7 Hz, 2H, C*H*_2_), 1.37 (t, *J* = 7 Hz, 3H, C*H*_3_). ^19^F NMR (376 MHz, CDCl_3_/CD_3_OD = 1:1): *δ*_F_ (ppm) = −105.08 (pdd, *J* = 7 Hz, *J* = 14 Hz, 1x*F*), −105.89 to −106.04 (m, 1×*F*). ^13^C NMR (101 MHz, CDCl_3_/CD_3_OD = 1:1): *δ*_C_ (ppm) = 179.8, 170.4, 165.4 (dd, *J* = 12 Hz, *J* = 254 Hz), 165.0 (d, *J* = 6 Hz), 161.9 (dd, *J* = 12 Hz, *J* = 260 Hz), 141.1, 137.9, 132.7 (dd, *J* = 4 Hz, *J* = 10 Hz), 130.5, 126.4, 123.5, 123.4, 120.9, 120.2, 119.2, 113.4, 112.6 (dd, *J* = 4 Hz, *J* = 22 Hz), 112.1 (dd, *J* = 4 Hz, *J* = 13 Hz), 109.2, 109.0, 105.6 (t, *J* = 26 Hz), 38.0, 32.9, 22.8, 14.0. HR ESI +MS: C_25_H_20_F_2_N_4_O_2_, *m*/*z* = 447.16279 [M+H]^+^, 469.14455 [M+Na]^+^, 893.31771 [2M+H]^+^, 915.29946 [M+Na]^+^.

### 3-(3-(4-Amino-2-fluorophenyl)-1,2,4-oxadiazol-5-yl)-*N*-(9-ethyl-9*H*-carbazol-3-yl)propanamide (6i)

Tin(II) chloride (40 mg, 211 μmol, 5.0 eq.) was added to deoxygenated **6d** (20 mg, 42 μmol, 1.0 eq.) in ethanol (1.0 mL), and the reaction mixture was stirred for 30 min at reflux. Saturated sodium carbonate solution (2.0 mL) was added at 0°C. The precipitate was filtered up, washed with water, diluted in chloroform/methanol (1:1), filtered and evaporated to dryness after concentration of the solvent *in vacuo*. The crude material was purified by column chromatography on silica gel (elution with chloroform/methanol 100:1) and by crystallization in the fridge (acetonitrile) to give **6i** (12 mg, 26 μmol, 62% yield) **6i**: ^1^H NMR (300 MHz, CD_3_CN, 65°C): *δ* (ppm) = 8.38 (s broad, 1H, N*H*), 8.32 (s, 1H, C*H*), 8.07 (d, *J =* 8 Hz, 1H, C*H*), 7.74 (t, *J =* 8 Hz, 1H, C*H*), 7.60 to 7.36 (m, 4H, 4×C*H*), 7.19 (t, *J =* 7 Hz, 1H, C*H*), 6.61 to 6.41 (m, 2H, 2×C*H*), 4.71 (s broad, 2 H, N*H*2), 4.39 (q, *J =* 7 Hz, 2H, C*H*_2_), 3.31 (t, *J =* 7 Hz, 2H, C*H*_2_), 2.96 (t, *J =* 7 Hz, 2H, C*H*_2_), 1.38 (t, *J =* 7 Hz, 3H, C*H*_3_). ^19^ F NMR (282 MHz, CD_3_CN, 65°C): *δ* (ppm) = −110.20 (dd, *J =* 9 Hz, *J =* 13 Hz). HRMS (ESI, positive ion) *m*/*z*: 466.16522 [M+Na]^+^, 909.34029 [2M+Na]^+^.

### 3-(3-(4-Fluoro-2-hydroxyphenyl)-1,2,4-oxadiazol-5-yl)-*N*-(9-ethyl-9*H*-carbazol-3-yl)propanamide (6j)

Compound **6j** was prepared using **19** as described in the synthesis of **6a**. The crude material was purified as described for **6h**, giving 38 mg of **6j** (85 μmol, 23% yield). **6j**: ^1^H NMR (300 MHz, CD_3_CN): *δ* (ppm) = 8.55 (s, broad, 1H, N*H*), 8.35 (d, *J =* 1 Hz, 1H, C*H*), 8.06 (pd, *J =* 6 Hz, 1H, C*H*), 8.00 (dd, *J =* 5 Hz, *J =* 7 Hz, 1H, C*H*), 7.55 to 7.42 (m, 4H, 4xC*H*), 7.18 (td, *J =* 1 Hz, *J =* 5 Hz, 1H, C*H*), 6.84 to 6.74 (m, 2H, 2xC*H*), 4.38 (q, *J =* 5 Hz, 2H, C*H*_2_), 3.37 (t, *J =* 5 Hz, 2H, C*H*_2_), 3.02 (t, *J =* 5 Hz, 2H, C*H*_2_), 1.35 (t, *J =* 5 Hz, 3H, C*H*_3_). ^19^F NMR (282 MHz, CD_3_CN): *δ* (ppm) = −108.31 to −108.44 (m). HR ESI +MS: C_25_H_21_FN_4_O_3_, *m*/*z* = 445.16724 [M+H]^+^, 467.14897 [M+Na]^+^, 889.32803 [2M+H]^+^, 911.30937 [2M+Na]^+^.

### 3-(3-(4-Fluoro-2-(2-fluoroethoxy)phenyl)-1,2,4-oxadiazol-5-yl)-*N*-(9-ethyl-9*H*-carbazol-3-yl)-propanamide (6k)

Potassium carbonate (14 mg, 101 μmol, 2.0 eq.) was added to deoxygenated **6j** (22 mg, 50 μmol, 1.0 eq.) in dry dimethylformamide (0.5 mL), and the reaction mixture was stirred for 5 min. To the reaction mixture, 1-fluoro-2-iodoethane (18 mg, 101 μmol, 2.0 eq.) was added, and the reaction mixture was stirred for 20 h at 50°C. The reaction mixture was added to ice water, washed with ethyl acetate and evaporated to dryness after concentration of the solvent *in vacuo*. The crude material was purified by column chromatography on silica gel (elution with petrol spirit or petrol ether/ethyl acetate 1:1) and by crystallization at room temperature (DCM/methanol) to give **6k** (11 mg, 22 μmol, 43% yield). **6k**: ^1^H NMR (300 MHz, CDCl_3_/CD_3_OD 1:1): *δ* (ppm) = 8.52 (s, broad, 1H, N*H*), 8.36 (d, *J =* 2 Hz, 1H, C*H*), 8.06 (pd, *J =* 8 Hz, 1H, C*H*), 7.94 (dd, *J =* 7 Hz, *J =* 9 Hz, 1H, C*H*), 7.58 to 7.39 (m, 4H, 4×C*H*), 7.18 (pt, *J =* 7 Hz, 1H, C*H*), 6.93 (dd, *J =* 2 Hz, *J =* 11 Hz, 1H, C*H*), 6.86 (dt, *J =* 2 Hz, *J =* 8 Hz, 1H, C*H*), 4.88 to 4.78/4.71 to 4.61 (dm, *J =* 48 Hz, 1 H/1H, C*H*_2_), (m, 1H, C*H*_2_), 4.47 to 4.32/4.31 to 4.22 (m, 2H, 1H/1H, 2×C*H*_2_), 3.31 (t, *J =* 7 Hz, 2H, C*H*_2_), 2.98 (t, *J =* 7 Hz, 2H, C*H*_2_), 1.35 (t, *J =* 7 Hz, 3H, C*H*_3_). ^19^F NMR (282 MHz, CDCl_3_/CD_3_OD 1:1): *δ* (ppm) = −108.79 to −108.96 (m, 1F), −223.78 to −224.42 (m, 1F). HR ESI +MS: C_27_H_24_F_2_N_4_O_3_, *m*/*z* = 513.17095 [M+Na]^+^, 1,003.35233 [2M+Na]^+^.

### 3-(3-(4-Fluoro-2-methoxyphenyl)-1,2,4-oxadiazol-5-yl)-*N*-(9-ethyl-9*H*-carbazol-3-yl)-propanamide (6 l)

Compound **6l** was synthesized via coupling method A as described for **6a** with **20** as carboxylic acid. The crude material was purified following the procedure for **6e**, yielding **6l** (76 mg, 166 μmol, 57%). **6l**: ^1^H NMR (400 MHz, CDCl_3_/CD_3_OD = 1:1): *δ*_H_ (ppm) = 8.29 (d, *J* = 2 Hz, 1H, C*H*), 8.02 to 7.91 (m, 2H, 2×C*H*), 7.48 (dd, *J* = 2 Hz, *J* = 8 Hz, 1H, C*H*), 7.43 to 7.33 (m, 2H, 2×C*H*), 7.30 (d, *J* = 9 Hz, 1H, C*H*), 7.13 (pt, *J* = 7 Hz, 1H, C*H*), 6.79 (dd, *J* = 2 Hz, *J* = 11 Hz, 1H, C*H*), 6.72 (dd, *J* = 2 Hz, *J* = 8 Hz, 1H, C*H*), 4.29 (q, *J* = 7 Hz, 2 H, C*H*_2_), 3.88 (s, 3H, C*H*_3_), 3.35 (t, *J* = 7 Hz, 2H, C*H*_2_), 3.01 (t, *J* = 7 Hz, 2H, C*H*_2_), 1.34 (t, *J* = 7 Hz, 3H, C*H*_3_). ^13^C NMR (101 MHz, CDCl_3_/CD_3_OD = 1:1): *δ*_C_ (ppm) = 178.7, 170.5, 166.6, 166.2 (d, *J* = 251 Hz), 160.4 (d, *J* = 11 Hz), 141.1 137.8, 133.3 (d, *J* = 11 Hz), 130.5, 126.4, 123.5, 123.4, 120.9, 120.1, 119.2, 113.4, 112.3 (d, *J* = 3 Hz), 109.2, 109.0, 108.0 (d, *J* = 22 Hz), 100.5 (d, *J* = 26 Hz), 56.4, 38.0, 33.0, 22.8, 14.0. HR ESI +MS: C_26_H_23_FN_4_O_3_, *m*/*z* = 459.18247 [M+H]^+^, 917.35797 [2M+H]^+^.

### 3-(3-(2-Bromo-4-methoxyphenyl)-1,2,4-oxadiazol-5-yl)-*N*-(9-ethyl-9*H*-carbazol-3-yl)propanamide (6m)

Compound **6m** was synthesized as **6a** with **14** as carboxylic acid reactant. The crude material was purified by column chromatography on silica gel (elution with DCM/methanol 100:1 and DCM/methanol 100:2) and by crystallization at room temperature (DCM/methanol), yielding **6m** (83 mg, 159 μmol, 54%). **6m**: ^1^H NMR (400 MHz, CDCl_3_/CD_3_OD = 1:1): *δ*_H_ (ppm) = 8.29 (s, 1H, C*H*), 8.00 (d, *J* = 8 Hz, 1H, C*H*), 7.72 (d, *J* = 8 Hz, 1H, C*H*), 7.55 to 7.27 (m, hidden by solvent, 4×C*H*), 7.22 (d, *J* = 2 Hz, 1H, C*H*), 7.15 (t, *J* = 7 Hz, 1H, C*H*), 6.92 (dd, *J* = 2 Hz, *J* = 9 Hz, 1H, C*H*), 4.32 (q, *J* = 7 Hz, 2H, C*H*_2_), 3.80 (s, 3H, C*H*_3_), 3.38 (t, *J* = 7 Hz, 2H, C*H*_2_), 3.02 (t, *J* = 7 Hz, 2H, C*H*_2_), 1.36 (t, *J* = 7 Hz, 3H, C*H*_3_). ^13^C NMR (101 MHz, CDCl_3_/CD_3_OD = 1:1): *δ*_C_ (ppm) = 179.4, 170.4, 168.2, 162.4, 141.0, 137.8, 133.3, 130.5, 126.4, 123.4, 123.3, 123.2, 120.9, 120.7, 120.13, 120.09, 119.2, 113.9, 113.4, 109.1, 109.0, 56.0, 38.0, 32.9, 22.8, 14.0. HR ESI +MS: C_26_H_23_BrN_4_O_3_, *m*/*z* = 519.10249 [M+H]^+^, 541.08500 [M+Na]^+^, 1,037.19973 [2M+H]^+^, 1,059.18045 [2M+Na]^+^.

### 3-(3-(2,4-Difluorophenyl)-1,2,4-oxadiazol-5-yl)-*N*-(1-benzyl-1*H*-indol-3-yl)-propanamide (7h)

Compound **7h** was prepared by coupling of **16** with 1-benzyl-1*H*-indol-3-amine hydrochloride according to coupling method A. The crude product was purified by column chromatography on silica gel (elution with petrol spirit/ethyl acetate 2:1 and ethyl acetate), yielding **7h** (71 mg, 155 μmol, 87%). For affinity data, the product (20 mg) was further purified by HPLC (RP18, ReproSil-Pur 120 ODS3, endc., acetonitrile/0.057 M triethylammonium acetate = 65:35) and by crystallization at room temperature (ethanol/water) to an aspic. The aspic was dried in a speed vacuum to give 14.7 mg of pure solid product. **7h**: ^1^H NMR (400 MHz, CDCl_3_): *δ*_H_ (ppm) = 8.07 to 7.98 (m, 1H, C*H*), 7.92 (s broad, 1H, N*H*), 7.78 (s, 1H, C*H*), 7.51 (d, *J* = 8 Hz, 1H, C*H*), 7.36 to 7.22 (m, hidden by solvent, 4×C*H*), 7.19 (pt, *J* = 7 Hz, 1H, C*H*), 7.16 to 7.10 (m, 2H, 2×C*H*), 7.07 (pt, *J* = 7 Hz, 1H, C*H*), 7.02 to 6.92 (m, 2H, 2×C*H*), 5.24 (s, 2H, C*H*_2_), 3.42 (t, *J* = 7 Hz, 2H, C*H*_2_), 3.06 (t, *J* = 7 Hz, 2H, C*H*_2_). ^19^F NMR (376 MHz, CDCl_3_): *δ*_F_ (ppm) = −104.01 to −104.26 (m, 1x*F*), −104.97 to −105.20 (m, 1x*F*). ^13^C NMR (101 MHz, CDCl_3_): *δ*_C_ (ppm) = 178.8, 167.7, 164.7 (dd, *J* = 12 Hz, *J* = 254 Hz), 164.6 (d, *J* = 6 Hz), 161.3 (dd, *J* = 12 Hz, *J* = 260 Hz), 137.4, 134.1, 132.1 (dd, *J* = 4 Hz, *J* = 10 Hz), 128.9, 127.8, 127.0, 122.6, 121.1, 119.9, 119.3, 116.8, 114.1, 112.1 (dd, *J* = 4 Hz, *J* = 22 Hz), 111.6 (dd, *J* = 4 Hz, *J* = 13 Hz), 110.1, 105.3 (t, *J* = 25 Hz), 50.3, 32.6, 22.6. HR ESI +MS: C_26_H_20_F_2_N_4_O_2_, *m*/*z* = 481.14447 [M+Na]^+^, 939.29960 [2M+Na]^+^.

### X-ray structural analysis

The single-crystal X-ray diffraction data of compound **6f** were collected on a diffractometer IPDS-2T (Stoe & Cie GmbH, Darmstadt, Germany) at 180 K using Mo-K_α_ radiation (*l* = 71.073 pm). The data reduction was performed using the STOE X-AREA software package (2006, Stoe & Cie, Darmstadt, Germany). The structure was solved by direct methods using the program SHELXS-97 and refined using SHELXL-97 [46]. All non-H atoms were refined anisotropically, and hydrogen (H) atoms were added geometrically using a riding model. Graphical visualization of the structure was performed using the program Diamond 3.2e (DIAMOND 3.2e, K. Brandenburg, 2010, Crystal Impact GbR, Bonn, Germany). The crystal structure has been deposited at the Cambridge Crystallographic Data Centre and allocated the deposition number CCDC 841516.

### Cell culture

CHO cell lines stably transfected with human CB1 (hCB1-CHO; obtained from Euroscreen, Gosselies, Belgium) or human CB2 (hCB2-CHO; obtained from Paul L. Prather, Department of Pharmacology and Toxicology, College of Medicine, University of Arkansas for Medical Sciences, USA) were cultured in Ham's F12 medium or Dulbecco's modified Eagle's medium (DMEM) with 10% fetal calf serum, 100 IU/mL penicillin, 100 μg/mL streptomycin and 250 or 400 μg/mL of the selection antibiotic Geneticin (G418), respectively. The cells were cultured in a humidified atmosphere with 5% CO_2_ at 37°C. Confluent cell layers of 175-cm^2^ flasks were scraped; cells were harvested by centrifugation (800 rpm, 3 min), counted by trypan blue staining, suspended in 200 μL of 50 mM tris(hydroxymethyl)aminomethane (TRIS; pH 7.4) at 4°C and stored at −32°C until the binding experiment.

### Determination of CB receptor affinity

Competitive binding experiments were performed with crude membrane homogenates obtained from hCB1-CHO or hCB2-CHO cells as described above. Increasing concentrations of test compounds (0.01 nM to 10 μM) diluted from 10 mM stock solutions in DMSO were incubated with 30 to 50 μg of membrane protein (equivalent to 0.5 to 1 × 10^6^ hCB1-CHO or hCB2-CHO cells) and 0.5 nM [^3^H]CP55,940 (6,438 GBq/mmol; PerkinElmer Life and Analytical Sciences, Rodgau, Germany) in a final volume of 1 mL in a binding buffer (50 mM TRIS (pH 7.4) at 21°C, 0.1% bovine serum albumin (BSA), 5 mM MgCl_2_, 1 mM EDTA) for 90 min at room temperature. Non-specific binding of [^3^H]CP55,940 was determined in the presence of 10 μM CP55,940. Incubations were terminated by rapid filtration through a GF-B glass fibre filter pre-incubated for 90 min at room temperature in freshly prepared 0.5% polyvinylpyrrolidone + 0.1% Tween 20 solution using a 48-well cell harvester (Brandel, Gaithersburg, MD, USA).

Saturation binding of CP55,940 towards hCB1-CHO or hCB2-CHO cell membranes was analysed in homologous competition experiments performed with increasing concentrations of CP55,940 (0.01 nM to 10 μM) and one concentration of [^3^H]CP55,940 (0.5 nM). Kinetic analysis of the binding of CP55,940 towards hCB2-CHO cell membranes was performed in one association experiment using three concentrations of [^3^H]CP55,940 (0.207, 0.458 and 0.873 nM) and incubation times of up to 120 min.

Binding data were analysed using GraphPad Prism version 2.01 (GraphPad Software, Inc., San Diego, CA, USA) by non-linear regression to provide estimates of the half maximal inhibitory concentration (IC_50_) values and the rate of association. *K*_D_ values of ^3^H]CP55,940 from homologous radioligand displacement experiments were estimated according to Jeffries et al. [47] by *K*_D_ = IC_50_ – [radioligand] and from kinetic experiments according to Hulme and Trevethick [48]. The corresponding inhibition constants (*K*_i_) of test compounds were derived from the IC_50_ values using the Cheng-Prusoff equation [49]. All binding experiments were performed in triplicates, and data are given as mean values from independent experiments.

### Animals

Animal experiments were performed under procedures approved by the State of Saxony Animal Care and Use Committee and conducted in accordance with the German Law for the Protection of Animals. Female CD-1 mice (10 to 12 weeks old, 20 to 25 g), obtained from the Medizinisch-Experimentelles Zentrum der Universität Leipzig (Leipzig, Germany), were used for the experiments.

### Autoradiographic binding study

The animals were anesthetized and sacrificed, and their spleens were rapidly removed and frozen in isopentane at −35°C. The spleens were brought to −15°C in a cryostat (Microm International GmbH, Walldorf, Germany), and a series of 12-μm-thick coronal sections obtained from the spleens of three animals were collected in parallel on microscopic glass slides. The sections were dried at room temperature and stored at −35°C until they were processed for receptor binding autoradiography.

For autoradiographic experiments, the slides were brought to room temperature, dried in a stream of cold air, pre-incubated in a binding buffer (50 mM TRIS (pH 7.4) at 21°C, 5% BSA) for 15 min at room temperature and dried again in a stream of cold air. To account for total binding, samples were incubated for 2 h at room temperature with 6 nM [^3^H]CP55,940 (5,328 GBq/mmol, PerkinElmer Life and Analytical Sciences, Rodgau, Germany). Sections were incubated in the presence of 1 μM CP55,940 (non-specific binding), SR144528 (CB2-selective inverse agonist), SR141716A (CB1-selective antagonist), or a test compound in binding buffer. Samples were washed twice for 30 min at 4°C in 50 mM TRIS (pH 7.4) at 4°C, containing 1% BSA, dipped briefly in ice-cold deionized water (5 s), dried in a stream of cold air and exposed along with [^3^H] standards (Amersham/GE Healthcare, Piscataway, NJ, USA) for 14 days to [^3^H]-sensitive screen plates (Fuji Photo Film, Co. Ltd., Tokyo, Japan).

The image plates were analysed using a BAS-1800II system bioimaging analyser (Fuji Photo Film, Co. Ltd., Tokyo, Japan). Scan data were visualized and processed by computer-assisted microdensitometry (Aida version 2.31, Raytest Isotopenmessgeräte GmbH, Straubenhardt, Germany). Irregular regions of interest (ROIs) were drawn on selected areas of the spleen labelled as red pulp (low-density binding) and white pulp (high-density binding) according to Lynn and Herkenham [18] and Massi et al. [39]. The intensity of the radioligand binding was assessed by measuring the background-corrected optical density in each ROI expressed as photostimulated luminescence per square millimetre The theoretical fractional occupancy (*f*) of ^3^H]CP55,940 and the reference and test compounds at CB2 was calculated using the Gaddum equation: *f*A = [A] / ([A] + *K*_D__A_(1 + [B]/*K*_D,B_)) [50].

### Receptor modelling

The receptor model was created employing the ‘Homology Modeling’ module of the program Molecular Operation Environment (MOE, version 2010.11, Chemical Computing Group Inc. Montreal, Canada). For this purpose, the sequence of the human CB2 [Swiss-Prot:P34972] [4] was subjected to a PDB search on the protein structure database implemented in MOE 2010.11 with the Z cut-off set to 2.0 while leaving the other parameters at their default values. The search suggested the human adenosine receptor A2a (hAA2R) as the template for the comparative modelling procedure. Since the recently published structure of the agonist-bound hAA2 receptor [43] is not included in the MOE protein database, the hAA2R structure [PDB:3EML] [51] was chosen as reference for the sequence alignment. The alignment of the human CB2 sequence with that of hAA2R is given as Additional files 1, 2, and 3. After downloading the 3D structure of [PDB:3QAK] [43] from the RCSB database, the structure was superimposed on the crystal structure 3eml. After removal of all lipid molecules, ions and water molecules, the residues between 209 and 222, which belong to lysozyme T4, were removed from the structure. In order to improve the modelling accuracy, the co-crystalized agonist UK-432097 of [PDB:3QAK] remained in the system during the modelling procedure. Out of ten independently generated models employing the default parameters of the Homology Modeling module, a consensus model was computed. To remove clashes and optimize the overall geometry of the protein, the system was subjected to an energy minimization employing the implemented AMBER89 force field using the default values. The quality of the model was verified by running the Protein Report module of MOE. After removal of the template ligand UK-432097, the apo receptor was energy-minimized to convergence, giving the final hCB2 model structure.

### Docking studies

The small compounds were built in MOE with subsequent geometry optimization based on the MMFF force field as incorporated in the program package with a distance-dependent dielectric constant. The residues of the proposed binding pocket were defined based on the Site Finder module of MOE. The docking site included residue Y190^5.39^ (numbering scheme according to Ballesteros and Weinstein [52]), which is known to be involved in ligand binding (*cf.*[53] and references cited therein). For the molecular docking studies, the program GOLD [54] (version 5) with parameters set to their default values was employed. All single bonds of the small ligands were allowed to rotate freely during the docking simulation. The side chains of the residues composing the binding pocket were treated as flexible, and the atoms of the protein backbone were kept at fixed positions. This approach is considered to represent the main characteristics of an induced fit [55]. The predicted poses were clustered by the GOLD software with a cut-off of 1.0 Å (heavy atoms only), giving representative geometries of the small molecules. For each of the compounds, all final poses were subjected to a further analysis with the program PostDock [45].

### Radiochemistry

The radiolabelling procedure was initiated by addition of K[^18^F]/K2.2.2 and K_2_CO_3_ to 2 mg of precursor in dry *N*,*N*-dimethylformamide under diffuse light and argon atmosphere (**6g**: 293 MBq [^18^F]fluoride, K2.2.2 (7.4 μmol/GBq) and K_2_CO_3_ (3.2 μmol/GBq); **6c**: 303.2 MBq [^18^F]fluoride, K2.2.2 (7.4 μmol/GBq) and K_2_CO_3_ (3.2 μmol/GBq)). The reaction mixtures were subjected to microwave radiation in closed vessels employing a Discover microwave synthesizer (CEM GmbH, Kamp-Lintfort, Germany) with the following scheme: heating to 60°C (30 s, 45 to 75 W with subsequent cooling to 20°C followed by three radiation cycles (100°C (60 s, 150 W); 20°C). Subsequently, 5 eq. SnCl_2_ was added and the reaction mixture was subjected to three heating cycles (120°C (60 s, 150 W); 20°C). The yields of the labelled compounds were determined by radio-TLC (EE/petrol ether = 2:1).

## Conclusions

Various *N*-aryl-3-((hetero)aromatic-oxadiazolyl-propionamides have been synthesized. Among those, 9-ethyl-9*H*-carbazole-substituted compounds showed the highest affinity towards CB2 and good selectivity against CB1 receptors. The study revealed strong relationships between the structure of the CB2 ligands and their CB2 affinity and selectivity. The data of this study indicate that a modification of the compounds at the amide moiety shows a stronger impact on binding and specificity of the compounds toward CB2 than variation at the (hetero)aromatic ring located at the 3-position of the oxadiazoles. Thus, introduction of functional groups for subsequent ^18^F labelling at this moiety is a promising strategy for the creation of CB2-selective radioligands suitable for (neuro)imaging by PET. Such imaging studies are currently in process at our laboratory.

## Endnotes

^a^Vendors of chemicals (details can be obtained from the authors upon request): ABCR GmbH (Karlsruhe, Germany), Activate Scientific GmbH (Prien, Germany), Alfa Aesar Johnson Matthey Forschungschemikalien GmbH (Karlsruhe, Germany), Apollo Scientific LTD (Cheshire, UK), AppliChem GmbH (Darmstadt, Germany), Carl Roth GmbH & Co. KG (Karlsruhe, Germany), Fisher Scientific GmbH (Schwerte, Germany), Green Chempharm, Inc. (Bardonia, NY, USA), Hande Sciences (Suzhou, China), Oakwood Products Inc. (West Columbia, SC, USA), Princeton BioMolecular Research (Munich, Germany), Sigma-Aldrich Chemie GmbH (Munich, Germany), VWR International GmbH (Darmstadt, Germany).

## Competing interests

The authors declare that they have no competing interests.

## Disclaimer

Some of the syntheses described here have partially been disclosed in the patent DE 10 2010 063 974 A1.

## Supplementary Material

Additional file 1 **Contains crystal data and structure refinement for compound X-ray structural data of 6f.** Crystal data: C_26_H_20_N_6_O_4_, M_m_ = 480.48 g mol^–1^, triclinic, space group $P\overset{-}{1}$ (no. 2), *a* = 766.4(1) pm, *b* = 1236.3(1) pm, *c* = 1278.1(2) pm, *α* = 109.04(1)°, *β* = 100.34(1)°, *γ* = 91.31(1)°, *V* = 1,121.8(2)·× 10^6^ pm^3^, *Z* = 2, *r*_calc_ = 1.423 g cm^–3^, *μ*(Mo-K_α_) = 0.10 mm^–1^, crystal size 0.32 × 0.08 × 0.07 mm^3^, *Q*_max_ = 50°, 6,766 reflections collected, 3,761 unique, *R*_int_ = 0.046, 2,382 observed reflections, 330 parameters, *R*1 = 0.043 [*I* > 2s(*I*)], *wR*2 = 0.082 (all data), maximum and minimum residual electron density 0.18 and –0.14 × 10^–6^ Å^–3^, respectively. Click here for file

Additional file 2 Contains the alignment of the sequence of hCB2 with hAA2R. Click here for file

Additional file 3 Contains the 3D coordinates of the modelled human cannabinoid receptor 2. Click here for file
