# Supplementary figures and images for: Cannabinoid receptor type 2 (CB2)-selective N-aryl-oxadiazolyl-propionamides: synthesis, radiolabelling, molecular modelling and biological evaluation
Source: Org Med Chem Lett. 2012 Oct 15;2:32. doi: 10.1186/2191-2858-2-32 (PMC3598492; doi:10.1186/2191-2858-2-32)

# CLUSTAL 2.0.12 MULTIPLE SEQUENCE ALIGNMENT

File: CB2R-3qak.ps

Page 1 of 1

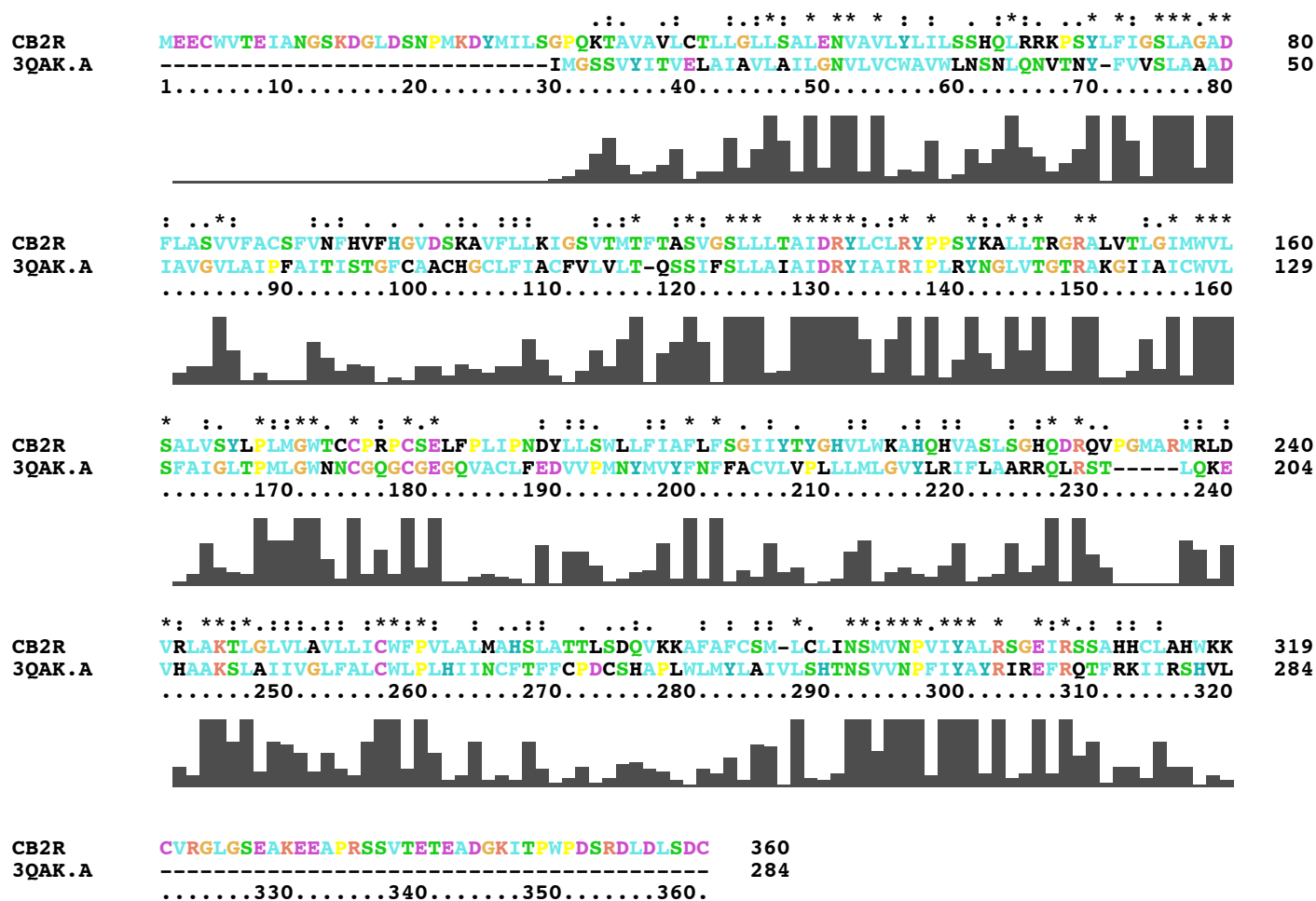

Supplement: Additional file 2 — Contains the alignment of the sequence of hCB2 with hAA2R. [file 2191-2858-2-32-S2.pdf]
